# Evaluating the establishment potential of cabbage stem flea beetle (Coleoptera: Chrysomelidae) and pollen beetle (Coleoptera: Nitidulidae) in canola-growing regions of North America using ensemble species distribution models

**DOI:** 10.1093/jee/toaf071

**Published:** 2025-04-16

**Authors:** Debra L Wertman, Vivek Srivastava, Tyler J Wist

**Affiliations:** Saskatoon Research and Development Centre, Agriculture and Agri-Food Canada, Saskatoon, Saskatchewan, Canada; Department of Forest and Conservation Sciences, University of British Columbia, Vancouver, British Columbia, Canada; Saskatoon Research and Development Centre, Agriculture and Agri-Food Canada, Saskatoon, Saskatchewan, Canada; Department of Forest and Conservation Sciences, University of British Columbia, Vancouver, British Columbia, Canada; Saskatoon Research and Development Centre, Agriculture and Agri-Food Canada, Saskatoon, Saskatchewan, Canada

**Keywords:** canola, oilseed rape, habitat suitability, invasion, climate change

## Abstract

Cabbage stem flea beetle, *Psylliodes chrysocephala* (Linnaeus 1758), and pollen beetle, *Brassicogethes viridescens* (Fabricius 1787), are pests of oilseed rape [*Brassica* spp. (Brassicales: Brassicaceae)] crops in Europe and pose a potential threat to canola production in North America. We used species occurrence and environmental data to develop ensemble species distribution models describing *P. chrysocephala* and *B. viridescens* habitat suitability, creating risk maps for either species under current (1981–2010; globally) and future [2011–2040 and 2041–2070, across 2 IPCC Shared Socio-economic Pathways (SSPs); North America only] environmental conditions. Projections for both species show improvement in northern North American habitat suitability under either SSP over time. Crop dominance was the most important predictor of suitable habitat for both species, followed by mean annual temperature range, precipitation metrics, and elevation (*P. chrysocephala* only). Risk maps for *P. chrysocephala* show broad habitat suitability, increasing under future scenarios, for this insect if it becomes introduced to North America; however, a phenological mismatch between *P. chrysocephala*, which specializes on winter oilseed rape (WOSR) in Europe, and spring oilseed rape (SOSR) would likely inhibit the long-term persistence of this insect in central North America. For *B. viridescens*, which impacts SOSR in Europe and is present in northeastern North America, predictive maps show increased risk in discontinuous patches across central North America that improve in suitability over time. While SOSR-cropping systems in central North America are environmentally suitable for both *P. chrysocephala* and *B. viridescens*, the establishment potential of these species may depend upon future sowing practices.

## Graphical Abstract

**Graphical Abstract:**
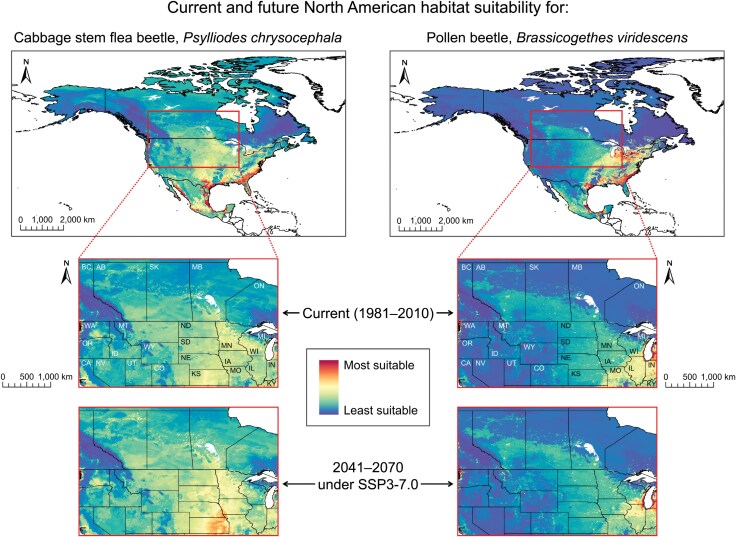

## Introduction

Invasive alien species pose enormous risks to agriculture and food security worldwide (Dosdall et al. 2011, Bradshaw et al. 2016, Paini et al. 2016, Skendžić et al. 2021a). Highly mobile insects that are introduced to novel habitats can be particularly destructive to agroecosystems. Feeding by herbivorous insects on the photosynthetic, conductive, and reproductive organs of cultivated plants can lead to injury, mortality, and disease transmission (Bradshaw et al. 2016, Skendžić et al. 2021a, Cornelsen et al. 2023). Future climate change is expected to exacerbate the impacts of native and invasive herbivorous pest insects on their host plants, as warming is already known to have influenced number of generations per year (voltinism), increased feeding and development rates, and led to range shifts in numerous insect species (Bajwa et al. 2020, Lehmann et al. 2020, Skendžić et al. 2021a, b). Furthermore, increased insecticide use associated with agricultural pest insects negatively affects biodiversity and natural enemy communities and has caused the evolution of insecticide resistance across a wide array of taxa (Forgash 1984, Stehle and Schulz 2015, Wood and Goulson 2017, Hladik et al. 2018). Defining the environmental parameters of habitat suitability for insect species with invasion potential can support monitoring and management programs that form the foundation of integrated pest management (IPM) in vulnerable agroecosystems (Skendžić et al. 2021b, Cornelsen et al. 2023).

Numerous insect pests feed upon and damage oilseed rape (*Brassica* spp.) crops throughout global growing regions (Gavloski et al. 2017, Zheng et al. 2020). Canadian oilseed rape crops are affected by multiple native and invasive insects that threaten crop establishment through the consumption of cotyledon and seedling vegetative tissue and, later in the growing season, reduce yields by feeding directly on buds and flowers (Gavloski 2017, Cornelsen et al. 2023). Impacts by these insects can result in crop failures, yield reductions, and steep management costs (Dosdall et al. 2011, Gavloski 2017, Cornelsen et al. 2023). Canola, a Canadian-branded oil product of oilseed rape, comprises multiple cultivars of several species [*Brassica napus* L., *B. rapa* L., and *B. juncea* (L.) Czern.] and is generally sown in the spring throughout the Canadian Prairie provinces (Canola Council of Canada 2024) and Great Plains [United States of America (USA); U.S. Canola Association 2024]. Canola cultivation and processing are major industries in Canada, specifically in the Prairie provinces (Saskatchewan, Alberta, and Manitoba), generating $29.9 billion dollars in annual economic impact (inclusive of exports for human consumption, biofuel, industrial use, and animal feed).

The cabbage stem flea beetle, *Psylliodes chrysocephala* (Linnaeus 1758), and the pollen beetle (also known as the bronzed or rape blossom beetle), *Brassicogethes viridescens* (Fabricius 1787), are major univoltine pests of oilseed rape in Europe (Mason et al. 2003, Williams 2010, Noronha and Mason 2017, Ortega-Ramos et al. 2022, Li et al. 2024). *P. chrysocephala*, which exerts most of its damage on young seedlings of winter oilseed rape (WOSR, sown in late summer/autumn) in Europe (Williams 2010, Ortega-Ramos et al. 2022, Li et al. 2024), has not yet been intercepted in Canada, but *B. viridescens*, which feeds upon reproductive tissues of SOSR in Europe, is established in Eastern Canada and Maine (USA) and was first intercepted in 1947 in Nova Scotia (Hoebeke and Wheeler 1996, Mason et al. 2003, Majka and Cline 2006, Majka et al. 2008, Noronha and Mason 2017). Potential for establishment of *P. chrysocephala* and *B. viridescens* in canola-growing regions of central North America [i.e., the Canadian Prairies and Great Plains (USA)] is not well elucidated, yet the consequences of establishment of these pests could be devastating to the North American canola industry if impacts parallel or exceed those observed in Europe (Mason et al. 2003, Olfert and Weiss 2006, Ortega-Ramos et al. 2022, Cornelsen et al. 2023, Li et al. 2024).

Species distribution models (SDMs) that identify suitable habitat for alien insect species in areas susceptible to introductions are powerful tools for assessing risk from potential invaders (Barbet-Massin et al. 2018, Srivastava et al. 2021, Gómez‑Undiano et al. 2022). In correlative SDM development, climatic and other environmental variables describing the preferred niche of a specific insect species are extrapolated from existing occurrence data, thereby allowing for projection of habitat suitability across space (i.e., the potential invaded range) and time (e.g., under various climate change scenarios) (Srivastava et al. 2019). SDMs are thus valuable tools for addressing the Wallacean shortfall (lack of species geographical information) that confounds the study of species distributions (reviewed in Hortal et al. 2015). For introduced species, where low founding population densities are difficult to detect, SDM projections can identify areas at current and future risk of invasion and help direct resources for ground surveys (Acosta et al. 2016, Barbet-Massin et al. 2018, Skendžić et al. 2021a, Srivastava et al. 2021, Gómez‑Undiano et al. 2022). Robust SDMs can provide critical support to biovigilance against incursions of non-native pest insect species, and to IPM of established populations of these insects should early detection and eradication be unsuccessful. In this study, we aimed to (i) build global SDMs (i.e., habitat suitability models and emergent projection maps) for *P. chrysocephala* and *B. viridescens*, with a focus on canola-growing regions in North America, (ii) use these SDMs to determine important predictors for potential establishment and subsequent range shifts of *P. chrysocephala* and *B. viridescens* in North America under climate change, and (iii) to assess the possible impacts of *P. chrysocephala* and *B. viridescens* to Canadian Prairie canola cultivation. Our results will provide government agencies, agribusiness, and growers with vital spatial and temporal information for surveying for and managing pressure from these potential invasive pest insects on North American canola crops.

## Materials and Methods

To predict suitable habitat for *P. chrysocephala* and *B. viridescens* globally, with a focus on canola-growing regions of North America, we created SDMs using publicly available species occurrence and environmental data. SDMs were constructed for the current period (1981–2010) and projected across 2 future periods (of 30-year intervals, 2011–2040 and 2041–2070) and 2 climate change scenarios [i.e., Shared Socio-economic Pathways (SSPs) from the Intergovernmental Panel on Climate Change (IPCC)]. The SSPs considered in our models were SSP1-2.6 [the sustainable (i.e., low emissions) scenario, with global development and climate protection measures compatible with a 2 °C warming target] and SSP3-7.0 [the upper-middle (i.e., high emissions) scenario, with global development consistent with 4 °C warming] (Pedersen et al. 2022, IPCC 2023). We chose the GFDL (Geophysical Fluid Dynamics Laboratory) Earth System Model Version 4.1 (GFDL-ESM 4.1; Dunne et al. 2020) as the general circulation model (GCM) of physical climate processes, from which we obtained the predicted values of each selected bioclimatic variable (see “Environmental variables” section). This GCM is also used to produce future climate grid projections across North America in Climate BC/WNA/NA models (Wang et al. 2016).

### Occurrence data for model development

**Fig. 1.**
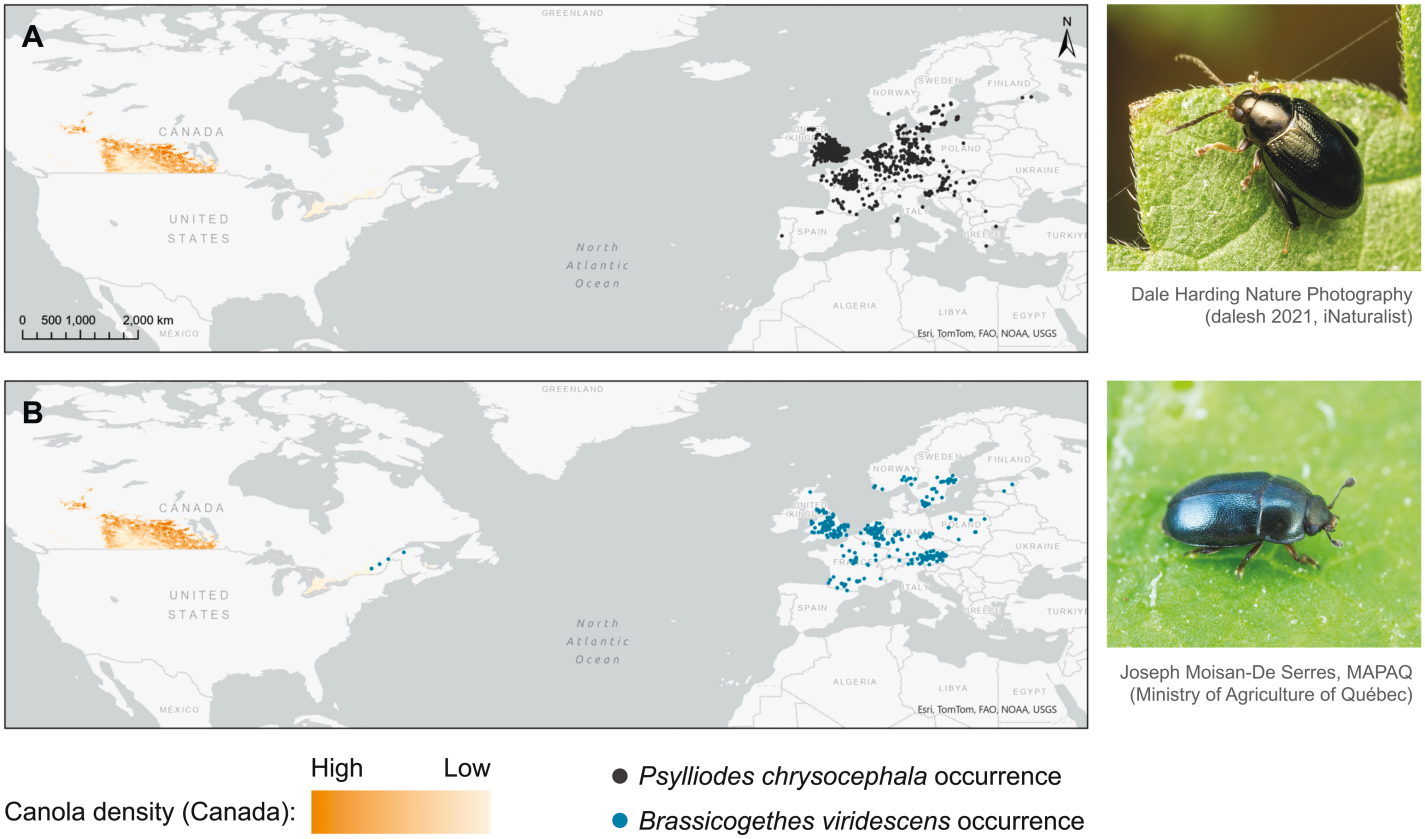
Maps of occurrences and images of A) the cabbage stem flea beetle, *Psylliodes chrysocephala*, and B) the pollen beetle, *Brassicogethes viridescens*, in Europe and North America. Occurrence data were obtained from the Global Biodiversity Information Facility (GBIF 2024a, b), the Centre for Agriculture and Bioscience International PlantwisePlus Knowledge Bank (for *P. chrysocephala*) (CABI 2021), and Agriculture and Agri-Food Canada records [AAFC, Christine Noronha (for *B. viridescens*)]. The spatial density of canola cultivation in Canada (2009–2021) is also shown (layer from AAFC 2022). The image of *P. chrysocephala* was obtained from dalesh (2021).

Presence records for *P. chrysocephala* and *B. viridescens* were obtained from: (i) the Global Biodiversity Information Facility (GBIF) online database for species occurrences (GBIF 2024a, b); (ii) the Centre for Agriculture and Bioscience International (CABI) PlantwisePlus Knowledge Bank (for *P. chrysocephala*) (CABI 2021); and (iii) records for *B. viridescens* provided by Agriculture and Agri-Food Canada (AAFC; Christine Noronha). Duplicate and erroneous records were removed, and data were further rarefied such that each observation fell inside a separate 5 × 5 km grid cell, leading to a total of 781 and 315 distinct occurrence records for *P. chrysocephala and B. viridescens*, respectively (Fig. 1). Spatial filtering was performed, using the spThin version 0.2.0 package (Aiello-Lammens et al. 2015) in R (version 4.2.1; R Core Team 2024), to mitigate sampling bias and to improve the predictive accuracy of the models. Then, 1,000 pseudo-absence (PA) locations with 10 repetitions each were generated, within an area defined by a buffer of 110 km around each focal species occurrence, using a surface range envelope model from the biomod2 version 4.2-6-1 package (Thuiller et al. 2009, 2024) in R. The buffer radius of 110 km was based upon the maximum daily dispersal distance of a well-studied forest pest, the mountain pine beetle, *Dendroctonus ponderosae* (Jackson et al. 2008, Srivastava and Carroll 2023). This surface range envelope model was used to randomly select PA outside this first envelope, i.e., in environmental conditions (determined by explanatory variables) that differed, within a proportionally standardized range, from those of presence points.

### Environmental Variables for Model Development

Environmental variables for modeling suitable habitat for *P. chrysocephala* and *B. viridescens* (Supplementary Table S1) were methodically selected from a set of 36 variables, which was assembled using existing knowledge of insect physiology and behavior. Environmental data were obtained for the locations of each occurrence of either species (see “Occurrence data for model development” section). For climate data, we obtained 32 bioclimatic variables [including Köppen–Geiger climate classification (Peel et al. 2007)] from the Chelsa database version 2.1 (Karger et al. 2017, 2021), at 1 km spatial resolution and averaged for the current period (1981–2010) and future periods under either climate scenario (2011–2040 and 2041–2070; SSP1-2.6 and SSP3-7.0). We considered additional biotic and abiotic variables that could affect the spatial responses of *P. chrysocephala* and *B. viridescens*, including host availability (i.e., crop dominance), elevation, human activity (Human Influence Index, HII), and soil quality (i.e., nutrient availability). Crop dominance data were sourced from NASA MEaSUREs Global Food Security Support Analysis Data [GFSAD, managed by NASA (National Aeronautics and Space Administration) and USGS (United States Geological Survey); Thenkabail et al. 2016]. Elevation data were derived from the 1-km resolution global 30 arc-s digital elevation model GTOPO30 (USGS 2018). Data for HII were acquired at 1-km resolution from the Socioeconomic Data and Applications Center (SEDAC) [WCS (Wildlife Conservation Society)/CIESIN (Center for International Earth Science Information Network, Columbia University) 2005]; HII is measure of direct human influence on terrestrial ecosystems derived from 9 global variables including human population density, land use, infrastructure, and land access that thereby functions as a metric for human-facilitated dispersal of insects. Soil quality data at 1-km resolution were obtained from the Harmonized World Soil Database v 1.2 (Fischer et al. 2008). All 36 environmental variables were resampled to 5 × 5 km grid resolution (and to 10 × 10 km grid resolution for global mapping under current conditions only).

To address multicollinearity among predictor variables, we performed variance inflation factor (VIF) analysis using the vifcor function within the usdm package (version 2.1-7; Naimi et al. 2014) in R. This pairwise correlation-based approach (sampling limit set at size = 5,000) identifies pairs of variables with the highest correlation exceeding a predefined threshold (0.7) and removes the variable with the larger VIF, iterating until no pairwise correlations exceed the threshold. The Pearson correlation coefficient (*r*) within usdm was used to quantify relationships among variables (Supplementary Fig. S1). Only variables with global coverage, VIF scores of < 10 after exclusion of collinear variables (Supplementary Table S2), and Pearson correlation coefficients (*r*) of |< 0.70| (Supplementary Fig. S1) were selected for inclusion in our models. This process resulted in 2 overlapping but distinct sets of 12 and 11 variables for modeling suitable habitat for *P. chrysocephala* and *B. viridescens*, respectively (Supplementary Table S1).

### Modeling and Model Training and Evaluation

**Fig. 2.**
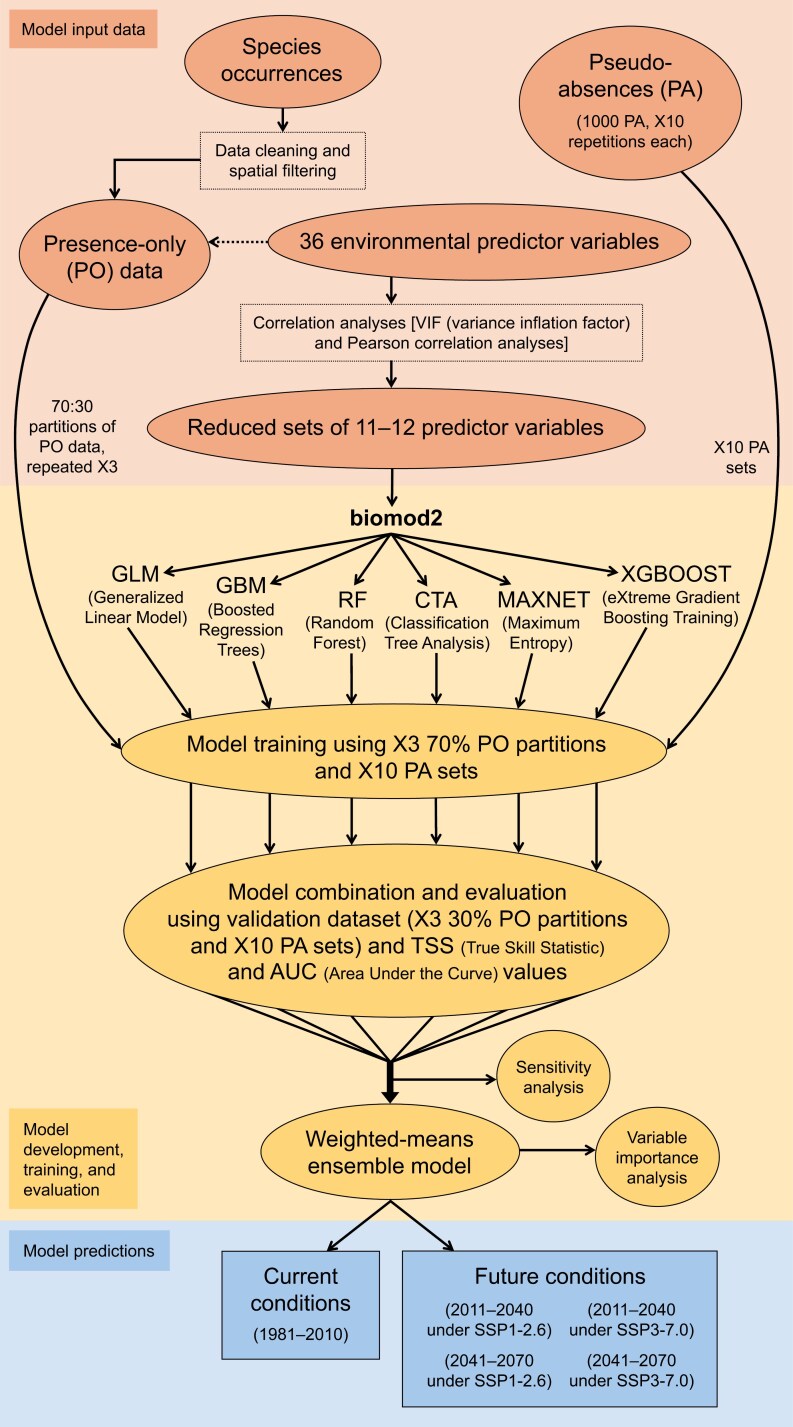
Schematic illustrating methods used for constructing the weighted-means ensemble species distribution models (SDMs) predicting habitat suitability for cabbage stem flea beetle, *Psylliodes chrysocephala*, and pollen beetle, *Brassicogethes viridescens*. Models were created using biomod2 version 4.2-6-1 (Thuiller et al. 2009, 2024) in RStudio version 2022.12.0 + 353 (R version 4.2.1; R Core Team 2024, RStudio Team 2024) and evaluated using True Skill Statistic (TSS; Allouche et al. 2006) values and area under the curve (AUC) values of the receiver operating characteristic (ROC) curve (Bradley 1997). See “Materials and Methods” section in the main text for additional details on species occurrence [presence-only (PO)] and pseudo-absence (PA) data, environmental predictor variable selection, model development, training, and evaluation, and sensitivity (i.e., uncertainty) and variable importance analysis.

To model *P. chrysocephala* and *B. viridescens* habitat suitability (see Fig. 2 for a visual summary of our methods), we first selected 6 regression and machine-learning-based SDM algorithms by assessing their compatibility and performance in previous studies featuring presence-only (PO) data supplemented by PAs (e.g., Srivastava et al. 2024). We then applied these model algorithms within an ensemble framework to predict suitable habitat for *P. chrysocephala* and *B. viridescens* under current conditions and future climate change scenarios. The package biomod2 version 4.2-6-1 (Thuiller et al. 2009, 2024) within RStudio version 2022.12.0 + 353 (R version 4.2.1; R Core Team 2024, RStudio Team 2024) was used to develop SDMs for *P. chrysocephala* and *B. viridescens*, implementing the selected algorithms: Generalized Linear Model (GLM; McCullagh and Nelder 1989), Boosted Regression Trees (i.e., Generalized Boosting Model, GBM; Ridgeway 1999, Friedman 2001), Random Forest (RF; Breiman 2001), Classification Tree Analysis (CTA; Breiman et al. 1984), Maximum Entropy [MAXNET; an alternative implementation of MAXENT (Phillips et al. 2006)], and eXtreme Gradient Boosting Training (XGBOOST; Chen and Guestrin 2016). Each model algorithm was run individually using the BIOMOD_Modeling() function and then fine-tuned using the BIOMOD_Tuning() function.

Occurrence (PO) data for *P. chrysocephala* and *B. viridescens* (see “Occurrence data for model development” section) were randomly divided into 70:30 ratios for each species where 70% of the data were used for model training and 30% were used for model evaluation. This division process was repeated 3 times (Gómez-Undiano et al. 2022). In total, 180 models were developed for each species, comprising 3 cross-validation runs using occurrence training data × 10 PA sets (see “Occurrence data for model development” section) × 6 model algorithms (= 180 models). Model combinations considered all single models (run + PA + algorithm) and the performance of each was evaluated using the full validation dataset [i.e., the union of presence (PO)-absence (PA) datasets] and True Skill Statistic (TSS; Allouche et al. 2006) values and area under the curve (AUC) values of the receiver operating characteristic (ROC) curve (Bradley 1997). TSS is a measure of the difference between success and error rates with values ranging from −1 to 1, where values ≤ 0 indicate that the performance of a model is not better than random, and a value of 1 indicates perfect performance (Allouche et al. 2006). The ROC curve describes the relationship between the proportion of observed presences predicted correctly (sensitivity) and the proportion of incorrectly predicted absences (1 minus specificity). A high (i.e., ≥ 0.7) AUC score, a metric of model performance that can range from 0 to 1, indicates that a model can accurately discriminate between locations at which the species is present or absent.

We constructed our final *P. chrysocephala* and *B. viridescens* habitat suitability models using a weighted ensemble approach, implementing the BIOMOD_EnsembleModeling() function to combine the best-performing models while accounting for algorithm biases and uncertainties associated with single forecasting methods. While all individual models for *P. chrysocephala* and *B. viridescens* were considered for ensemble modeling, only those with a TSS score ≥ 0.7 were selected so as to remove low-quality single models from either ensemble model. Ensemble predictions were generated for all 3 time periods (current and future) and both future SSPs using prob.mean.weight() inside the BIOMOD_Modeling() function, using the formula:


$$\begin{array}{l} WAprediction_{i}=\frac{\Sigma_{j}(TSS_{j}\times prediction_{ij}}{\Sigma_{j}TSS_{j}}, \end{array}$$ (1)

where for a given site, the WA ensemble prediction (WAprediction) is calculated as the sum of predictions for site across individual models, weighted by their respective TSS and TSS*j* values and normalized by the sum of all TSSs (Marmion et al. 2009). We forecasted future habitat suitability for either species using the BIOMOD_Projection() function to project either ensemble model onto future environmental data (see “Environmental variables for model development” section).

To assess uncertainty in our ensemble model predictions, we generated the coefficient of variation [standard deviation (SD)/mean] of probabilities over all selected individual models by converting continuous to binary data (TSS ≥ set threshold) for each time period and SSP. The threshold for converting continuous values into binary predictions was based upon the highest model evaluation (TSS) score, which indicates the probability above and below which species presence and absence is predicted. We also quantified shifts (expansion and contraction, in km^2^) in *P. chrysocephala* and *B. viridescens* habitat suitability by comparing the binary outputs (obtained as described above but for the ensemble model only) of current and future model predictions. The importance of each variable in predicting suitable habitat for *P. chrysocephala* and *B. viridescens* in either ensemble model was assessed using the variable.importance() function in biomod2: for each variable evaluated, the original variable was shuffled, model predictions were computed with the shuffled variable, and the Pearson correlation coefficient between the original and shuffled predictions was calculated. Variable importance scores were then calculated as 1 minus the correlation coefficient, where higher scores indicated greater model influence and scores of 0 represented no influence (without accounting for variable interactions).

### Mapping and Data Visualization

*P. chrysocephala* and *B. viridescens* occurrence maps, and maps showing current and future projections of *P. chrysocephala* and *B. viridescens* habitat suitability (using ensemble SDMs), were constructed in ArcGIS Pro (3.3.0; Esri Inc. 2024). A global map at 10-km resolution was produced for current period conditions, and projection maps at 5-km resolution were created for North America [whole continent and inset maps showing canola-growing regions of the Canadian Prairies and Great Plains (USA)] under current and future conditions (both time periods and both SSPs), for either species. Parallel sets of sensitivity maps showing coefficients of variation for model predictions (i.e., estimate certainty) were also produced for each species. The performance of each SDM algorithm and the ensemble models, and the importance of each environmental variable in either ensemble model, were visualized using ggplot2 (Wickham 2016) in RStudio version 2024.04.1 + 748 (R version 4.4.0; R Core Team 2024, RStudio Team 2024).

## Results

### Current and Future Global and North American Habitat Suitability for *P. chrysocephala*

**Fig. 3.**
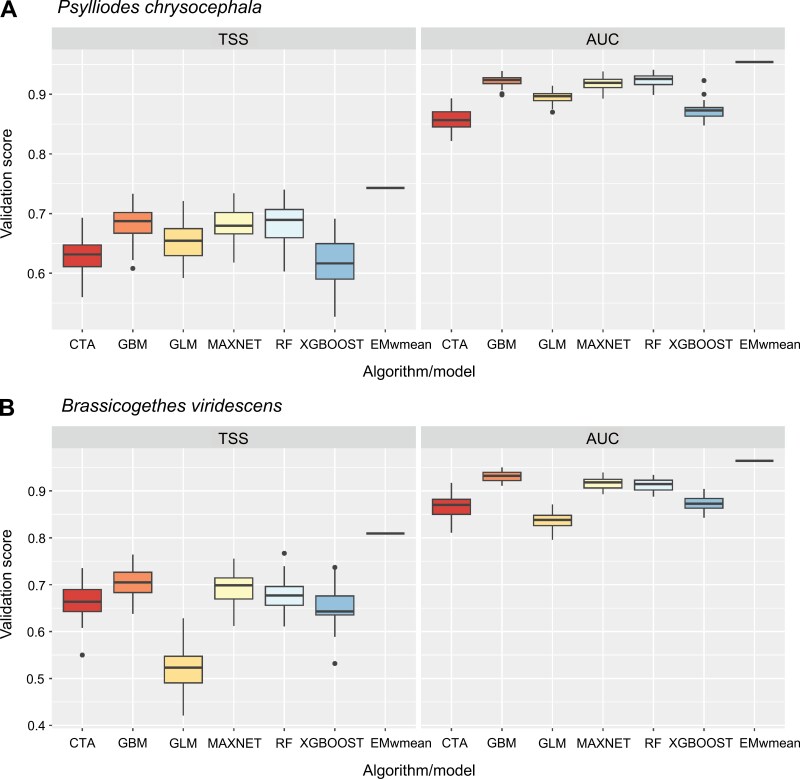
A) Performance [according to True Skill Statistic (TSS; Allouche et al. 2006) values and area under the curve (AUC) values of the receiver operating characteristic (ROC) curve (Bradley 1997)] of 7 species distribution models (SDMs) describing habitat suitability for cabbage stem flea beetle, *Psylliodes chrysocephala*, developed using 6 individual algorithms and a weighted-means ensemble (EMwmean) approach. B) Performance of 7 SDMs projecting current habitat suitability for the pollen beetle, *Brassicogethes viridescens*, generated using 6 individual algorithms and a weighted-means ensemble method. Horizontal lines across boxes indicate median model validation scores, upper and lower boxes represent the 75th and 25th quartiles, respectively, vertical lines show maximum and minimum values, and points indicate potential outliers.

Performance of individual models describing *P. chrysocephala* habitat suitability, which were combined (by weighted means) for the ensemble model, varied across algorithms (Fig. 3A; mean ± SD model evaluation scores to follow). GBM (TSS = 0.684 ± 0.028; AUC = 0.923 ± 0.010) and RF (TSS = 0.682 ± 0.035; AUC = 0.923 ± 0.010) performed best (i.e., having the highest evaluation scores), followed by MAXNET (TSS = 0.680 ± 0.031; AUC = 0.918 ± 0.011) and GLM (TSS = 0.655 ± 0.033; AUC = 0.895 ± 0.012). XGBOOST (TSS = 0.619 ± 0.038; AUC = 0.873 ± 0.015) and CTA (TSS = 0.629 ± 0.031; AUC = 0.858 ± 0.021) were the worst performing algorithms for forecasting *P. chrysocephala* habitat suitability. The weighted-means ensemble model for *P. chrysocephala* habitat suitability outperformed all individual models (TSS = 0.743; AUC = 0.954).

**Fig. 4.**
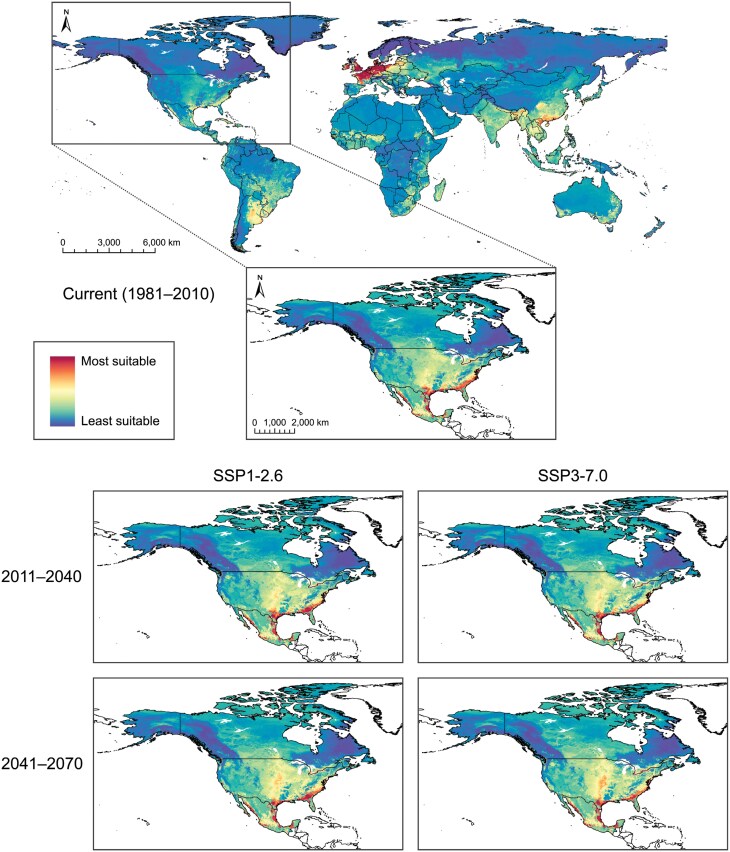
Habitat suitability (i.e., risk) maps for cabbage stem flea beetle, *Psylliodes chrysocephala*, based upon a weighted-means ensemble species distribution model (SDM), produced for current conditions (1981–2010; global at 10-km resolution, North America at 5-km resolution) and, for North America (at 5-km resolution), future climatic scenarios [SSP1-2.6 and SSP3-7.0, Shared Socio-economic Pathways (SSPs) from the Intergovernmental Panel on Climate Change (IPCC)] at 2 time periods (2011–2040 and 2041–2070).

**Fig. 5.**
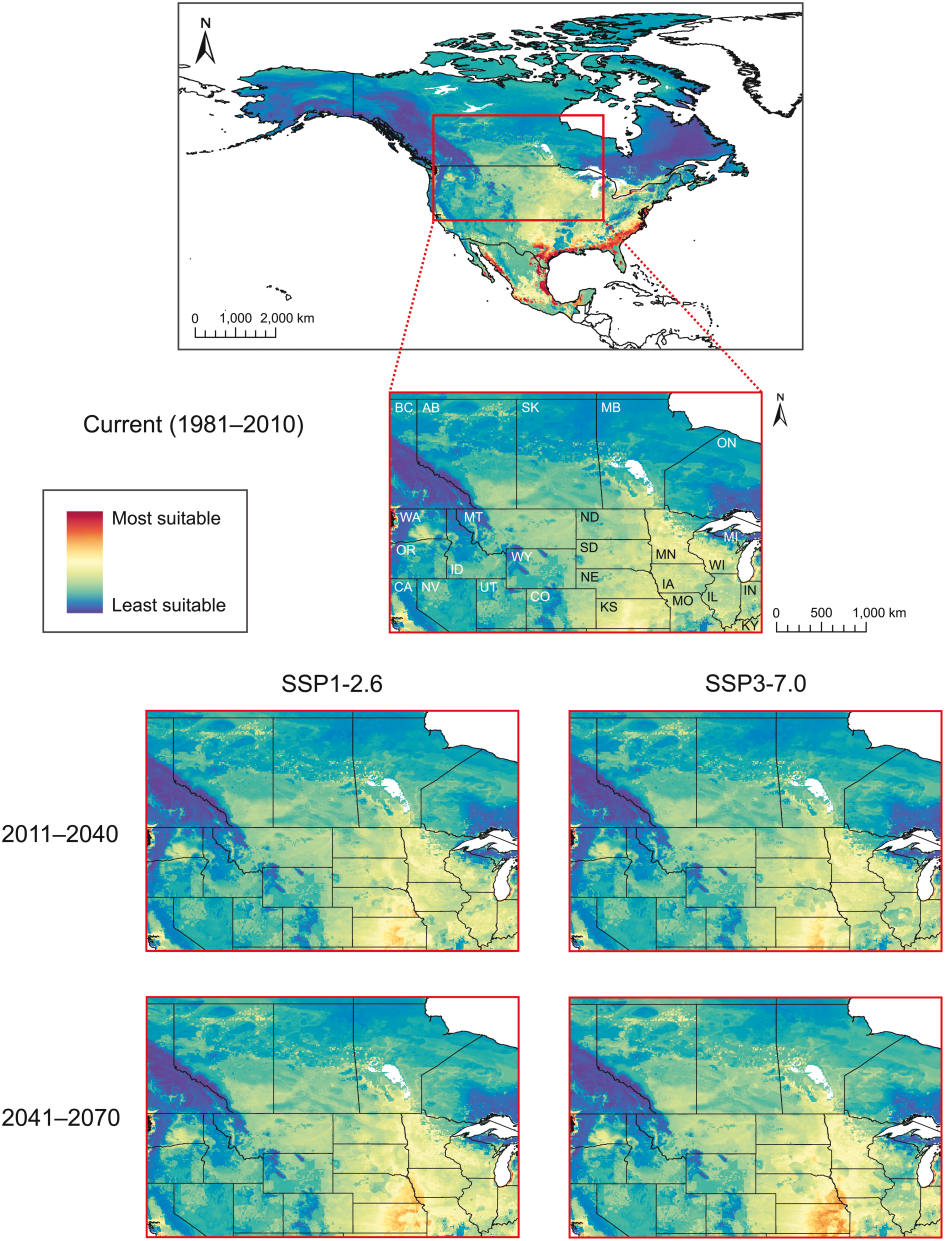
North American cabbage stem flea beetle, *Psylliodes chrysocephala*, habitat suitability maps (current condition map of North America from Fig. 4) featuring insets of Canadian Prairie and northern Great Plains (USA) regions at 5-km resolution under current (1981–2010; provinces and states labeled) and future climate change scenarios [SSP1-2.6 and SSP3-7.0, Shared Socio-economic Pathways (SSPs) from the Intergovernmental Panel on Climate Change (IPCC) at 2 time periods (2011–2040 and 2041–2070)].

Global projection of our *P. chrysocephala* ensemble model revealed moderate-to-high current habitat suitability (i.e., high degree of consensus among individual models) throughout the eastern and central USA, much of Mexico (including the eastern and western coasts), and Central America (Fig. 4). In South America, regions along the eastern coast (including eastern Bolivia) and throughout the western continent (including southern Brazil, eastern Paraguay, northeastern Argentina, and Uruguay) were also identified as having moderate-to-high current habitat suitability for *P. chrysocephala*. The *P. chrysocephala* model also showed high habitat suitability throughout Europe (particularly western Europe), eastern Russia, most of China (especially the northeast), Japan, Southeast Asia, Southern Asia (including India), and Madagascar. Patches of moderate-to-highly suitable habitat for *P. chrysocephala* were observed throughout Australia, New Zealand, and Africa. Prediction certainty was consistent with global habitat suitability for *P. chrysocephala*, with higher certainty in regions of higher suitability (Supplementary Fig. S2). For North America, the *P. chrysocephala* ensemble model indicated, with mixed certainty, high current habitat suitability in many parts of the USA and Mexico (particularly throughout the eastern coast of the USA, moderate-to-high certainty) (Fig. 4, Supplementary Fig. S2). Moderate-to-high current habitat suitability for *P. chrysocephala* was identified throughout southern Canada (east of British Columbia) with low-to-moderate certainty. Across the Canadian Prairies and northern Great Plains (USA), where the bulk of North American canola is grown, our model predicts moderate current habitat suitability, with large pockets of higher suitability, for *P. chrysocephala* (low to moderate certainty) (Fig. 5, Supplementary Fig. S3).

Under climate change in North America, our ensemble model predicted improvement in *P. chrysocephala* northern habitat suitability and an increase in overall suitability in the center of the continent (moderate-to-high certainty) (Figs. 4 and 5; Supplementary Figs. S2 and S3). Until the end of 2070, the area of most suitable habitat for *P. chrysocephala* (i.e., regions shaded red in Fig. 4) in North America is projected to shift under SSP1-2.6 (51,098.7 km^2^ expansion and 70,544.5 km^2^ contraction, with 112,005.9 km^2^ remaining unchanged) and SSP3-7.0 (34,244.3 km^2^ expansion and 100,248.5 km^2^ contraction, with 82,301.9 km^2^ remaining unchanged). With moderate certainty, the model predicted that *P. chrysocephala* habitat suitability in central North America will improve under future climatic conditions (Fig. 5, Supplementary Fig. S3). Projections indicated that most of the southern Canadian Prairies and Great Plains (USA) regions will become highly suitable, with some discontiguous patches of moderate suitability, for *P. chrysocephala* populations by 2041 to 2070 (under both SSPs).

Of the 12 environmental predictors selected for modeling *P. chrysocephala* habitat suitability (Supplementary Table S1), the strongest predictor [i.e., that with the highest variable importance (mean variable importance ± SD values to follow)] in the ensemble model was crop dominance (CD; 0.259 ± 0.005), followed by bio7 (0.112 ± 0.001), elevation (elev; 0.101 ± 0.003), bio15 (0.073 ± 0.001), bio2 (0.040 ± 0.000), bio19 (0.030 ± 0.000), bio9 (0.013 ± 0.000), bio18 (0.012 ± 0.000), HII (0.012 ± 0.000), Köppen–Geiger climate classification (kg2; 0.012 ± 0.000), nutrient availability (NA; 0.010 ± 0.000), and bio8 (0.008 ± 0.000) (Supplementary Fig. S4A). Response curves describing the relationship between each variable and ensemble model predictions are shown in Supplementary Fig. S4B; crop dominance strongly influenced *P. chrysocephala* habitat suitability, only showing a negative effect at the high and low extremes of the variable where crop cover was absent. *P. chrysocephala* habitat suitability was negatively associated with elevation, peaking at ~25 to 325 m and dropping steeply above ~325 m, and was weakly, positively influenced by HII. Regarding climate, regions with annual ranges of air temperature (bio7) of ~16 to 25 °C and precipitation seasonality (bio15) values ranging from ~10 to 17 kg/m^2^ were optimal for *P. chrysocephala*.

### Current and Future Global and North American Habitat Suitability for *B. viridescens*

The performance of individual models describing habitat suitability for *B. viridescens* also varied greatly by algorithm (Fig. 3B; mean ± SD model evaluation scores to follow). GBM (TSS = 0.705 ± 0.032; AUC = 0.931 ± 0.011) was the best-performing algorithm for *B. viridescens* habitat suitability (as for *P. chrysocephala*), followed by MAXNET (TSS = 0.692 ± 0.038; AUC = 0.916 ± 0.012) and RF (TSS = 0.677 ± 0.039; AUC = 0.912 ± 0.013). CTA (TSS = 0.665 ± 0.041; AUC = 0.866 ± 0.027), XGBOOST (TSS = 0.654 ± 0.044; AUC = 0.873 ± 0.016), and GLM (TSS = 0.522 ± 0.048; AUC = 0.838 ± 0.017) were the worst-performing algorithms for predicting *B. viridescens* habitat suitability. The weighted-means ensemble model performed better than each individual model in predicting *B. viridescens* habitat suitability (TSS = 0.809; AUC = 0.964).

**Fig. 6.**
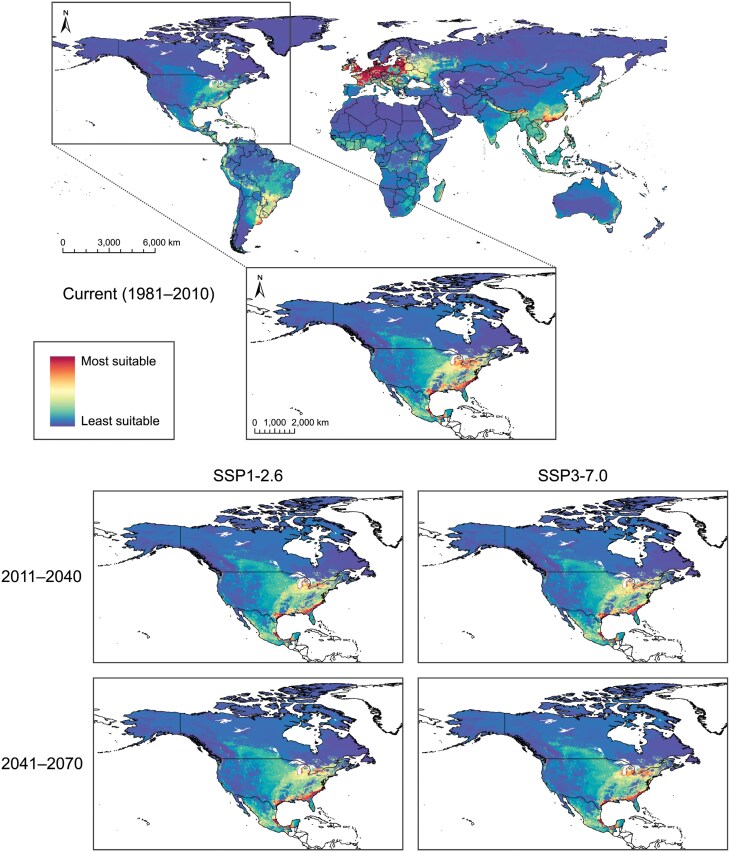
Habitat suitability maps for the pollen beetle, *Brassicogethes viridescens*, developed using a weighted-means ensemble species distribution model (SDM), for current conditions (1981–2010; global at 10-km resolution, North America at 5-km resolution) and, for North America (at 5-km resolution), future climatic scenarios [SSP1-2.6 and SSP3-7.0, Shared Socio-economic Pathways (SSPs) from the Intergovernmental Panel on Climate Change (IPCC)] at 2 time periods (2011–2040 and 2041–2070).

**Fig. 7.**
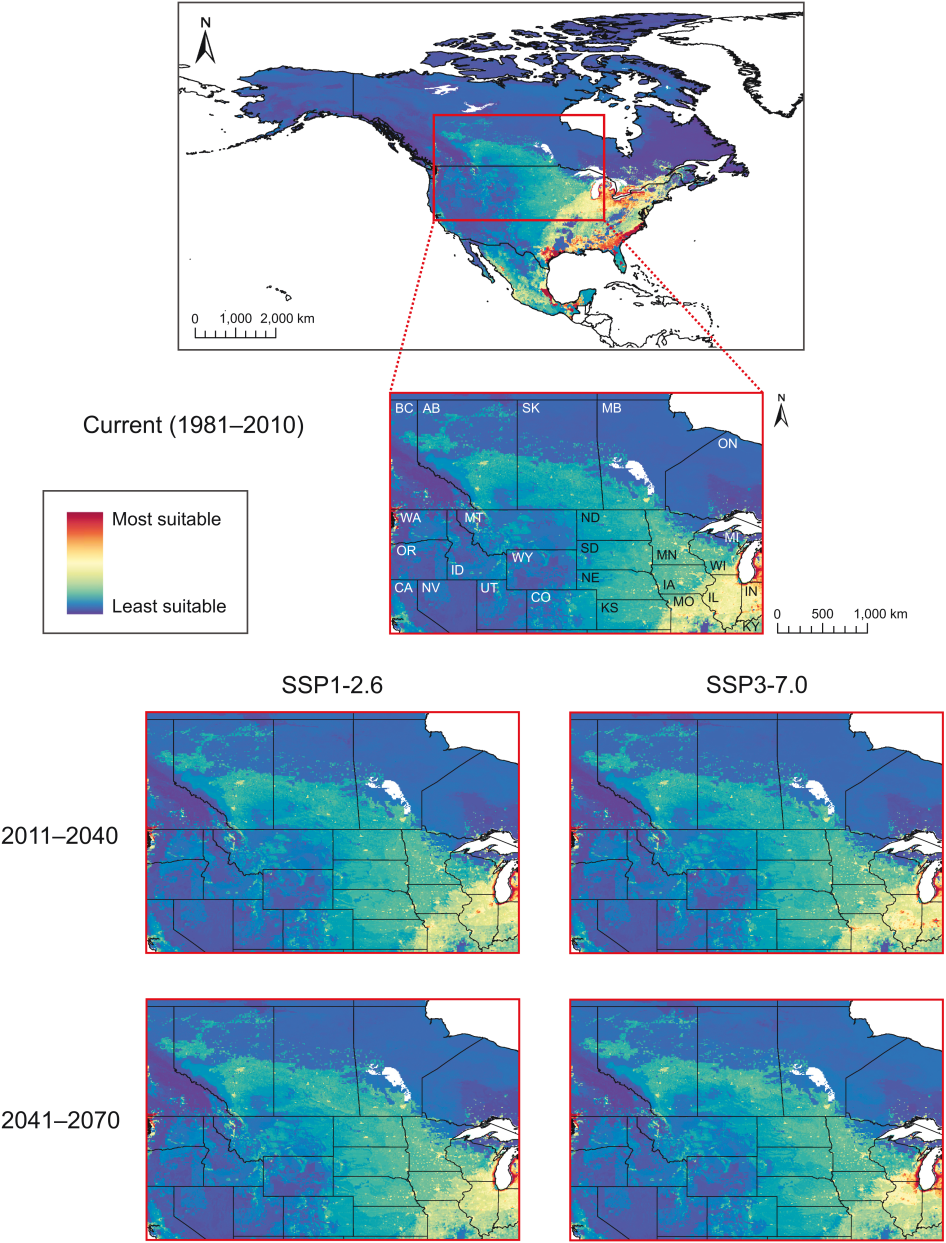
North American pollen beetle, *Brassicogethes viridescens*, habitat suitability maps (current condition map of North America from Fig. 6), showing insets of Canadian Prairie and northern Great Plains (USA) regions at 5-km resolution under current (1981–2010) and future climate change scenarios [SSP1-2.6 and SSP3-7.0, Shared Socio-economic Pathways (SSPs) from the Intergovernmental Panel on Climate Change (IPCC)] at 2 time periods (2011–2040 and 2041–2070).

Global projection of the *B. viridescens* ensemble model showed a geographic pattern similar to the *P. chrysocephala* projection but with less suitable habitat area and greater prediction certainty overall (Fig. 6, Supplementary Fig. S5). The model indicated moderate-to-high current habitat suitability for *B. viridescens* throughout the eastern USA, Mexico, and Central America. Moderate-to-high current habitat suitability for *B. viridescens* was also observed throughout much of South America, including countries along the western coast (Columbia, Ecuador, Peru, Bolivia, and southern Chile) and, in the east, southern Brazil, northeastern Argentina, eastern Paraguay, and Uruguay. High habitat suitability for *B. viridescens* was identified throughout Europe (especially Western Europe), eastern Russia, southeastern China, Japan, Southeast Asia, and along the eastern coasts of Australia and New Zealand. Moderate-to-high habitat suitability for *B. viridescens* was observed throughout South Asia (including India) and much of Africa (excluding northern Africa) and Madagascar. With moderate-to-high certainty, North American projection of the *B. viridescens* model showed high current habitat suitability in southeastern Canada (southern Ontario and Quebec and patches throughout the Maritime provinces, high certainty), and in the eastern USA and throughout Mexico (particularly the eastern coasts and Great Lakes regions, high certainty) (Fig. 6, Supplementary Fig. S5). Additionally, the model revealed large patches of moderate-to-low *B. viridescens* habitat suitability across the continent (moderate-to-high certainty). Central North America, including the Canadian Prairies and Great Plains (USA), exhibited low-to-moderate habitat suitability with small, scattered patches of higher habitat suitability (moderate-to-high certainty) (Fig. 7, Supplementary Fig. S6).

The model showed some increasing suitability of potential *B. viridescens* habitat at its northern extent, and reduction in southeastern habitat suitability, in North America under all climate change scenarios and time periods (moderate-to-high certainty) (Figs. 6 and 7, Supplementary Figs. S5 and S6). Until the end of 2070, the area of most suitable habitat for *B. viridescens* (i.e., shaded red on Fig. 6) is expected to shift under SSP1-2.6 (22,508.3 km^2^ expansion and 124,941.2 km^2^ contraction, with 256,393.0 km^2^ remaining unchanged) and SSP3-7.0 (34,437.0 km^2^ expansion and 132,179.9 km^2^ contraction, with 249,154.3 km^2^ remaining unchanged) across North America. With moderate-to-high certainty and small patches of low certainty, the Canadian Prairies region and eastern Great Plains (USA) will become more suitable for *B. viridescens* in the future, including an increase in the suitability of discontiguous habitat patches (Fig. 7, Supplementary Fig. S6). By 2041 to 2070, the model predicts reduction of habitat suitability for *B. viridescens*, except coastal regions, throughout the eastern USA (and to a lesser extent, southern Ontario, Quebec, and the Maritime provinces) under both SSPs (moderate-to-high certainty) (Fig. 6, Supplementary Fig. S5).

Of the 11 environmental variables used to model *B. viridescens* habitat suitability (Supplementary Table S1; mean variable importance ± SD values to follow), CD (0.799 ± 0.016) was the most important predictor in the ensemble model (as for *P. chrysocephala*) (Supplementary Fig. S7A). Also, like results for *P. chrysocephala*, bio7 (0.213 ± 0.003) was the second most important variable predicting habitat suitability for *B. viridescens*, followed by bio19 (0.208 ± 0.004), bio18 (0.094 ± 0.002), bio8 (0.093 ± 0.003), and bio14 (0.055 ± 0.001). HII (0.039 ± 0.001) and elevation (elev; 0.028 ± 0.001) were of low importance in predicting *B. viridescens* habitat suitability, followed by bio2 (0.028 ± 0.000), kg2 (0.005 ± 0.000), and NA (0.002 ± 0.000). Model response curves for each variable are shown in Supplementary Fig. S7B; *B. viridescens* habitat suitability was strongly associated with crop dominance, showing a similar pattern to the *P. chrysocephala* model where the relationship was negative at the lower and upper extents of the variable where crops were absent. For climatic predictors, annual ranges of air temperature (bio7) of ~16 to 29 °C and mean monthly precipitation amounts of the coldest quarter (bio19) ranging from ~11 to 25 kg/m^2^ per month positively influenced *B. viridescens* habitat suitability. The relationship between HII and *B. viridescens* habitat suitability was weakly positive.

## Discussion

The weighted-means ensemble models we developed for *P. chrysocephala* and *B. viridescens* showed strong predictive capacity for identifying suitable habitat for both species, performing far better than each individual model algorithm and exemplifying the utility of ensemble approaches to species distribution modeling (e.g., Watts and Worner 2008, Grenouillet et al. 2011, Srivastava et al. 2024). Our results showed that current global habitat suitability for both *P. chrysocephala* and *B. viridescens* largely overlaps with major oilseed rape cultivation regions in North America, Europe, Russia, China, India, Australia, and South America (Kirkegaard et al. 2021, AAFC 2022, U.S. Canola Association 2024), and that based upon habitat suitability alone, these regions may all be vulnerable to establishment of either species pending introduction and spread of founding individuals/populations. Habitat suitability for *P. chrysocephala* and *B. viridescens* in northern North America is projected to improve over time (assessed until 2070) under either climate change scenario (SSP). This study marks the first assessment of North American habitat suitability for *P. chrysocephala*, and our results for *B. viridescens* generally overlap with previous CLIMEX model predictions of habitat suitability for this species in Canada (Mason et al. 2003, Olfert and Weiss 2006). The *B. viridescens* ensemble model presented herein is more robust (i.e., comprising a suite of biotic and abiotic variables within an ensemble framework) and of higher resolution than these prior climate-only models, which show more extensive suitable area and extreme improvement of northern suitability under future conditions (3 °C temperate increase, no change in precipitation; Olfert and Weiss 2006) compared to our projections. The potential future utility of the models we present is reinforced by the demonstrated successes (i.e., validated by extraneous survey data) of other SDMs, developed using similar methods to ours, in predicting the distributions of both native [e.g., the Poweshiek skipperling, *Oarisma poweshiek* (Westwood et al. 2020); *Atyphella* spp. fireflies (Sutherland et al. 2021); and the dragonflies *Gomphurus ozarkensis* and *Somatochlora ozarkensis* (Boys et al. 2021)] and non-native [e.g., the bumblebee, *Bombus terrestris* (Acosta et al. 2016); Asian hornet, *Vespa velutina nigrithorax* (Barbet-Massin et al. 2018); and the African armyworm, *Spodoptera exempta* (Gómez‑Undiano et al. 2022)] insect species. These models thus succeeded in minimizing the Wallacean shortfall for each respective taxon.

Crop dominance was the most important predictor of both *P. chrysocephala* and *B. viridescens* habitat suitability, consistent with the tight association between these insects and their brassicaceous hosts. Both *P. chrysocephala* and *B. viridescens* are specialists in plants of the Brassicaceae family (and for *P. chrysocephala*, a few species from outside the Brassicaceae), including *Brassica* spp. oilseed rape/canola crops (*B. napus* and *B. rapa*) and brassicaceous weeds (such as *Sinapis* and *Raphanus* spp.) that grow abundantly on agricultural land (Majka et al. 2008, Warwick 2011, CABI 2021, Li et al. 2024). Interestingly, anthropogenic impact on the landscape (HII) was not important in defining habitat suitability for either *P. chrysocephala* or *B. viridescens*, suggesting that crop dominance alone sufficiently accounted for the role of human activity in the creation of suitable habitat for these species. It is therefore likely that dispersal corridors for these species are specifically linked to host plant availability as opposed to anthropogenic pathways, and that lower crop density across the western Great Lakes region might be a primary factor limiting the westward spread of *B. viridescens*, which is currently established in Eastern Canada (including Nova Scotia, Prince Edward Island, New Brunswick, and Québec) and Maine (USA) (Hoebeke and Wheeler 1996, Mason et al. 2003, Majka and Cline 2006, Majka et al. 2008).

The annual range of air temperature (bio7) was the second most important predictor of habitat suitability for both species, revealing that populations of *P. chrysocephala* and *B. viridescens* are strongly influenced by the difference between annual maximum and minimum temperatures. That both species require specific annual temperature ranges to successfully complete their life cycles reflects the minimum amount of heat accumulation needed to complete a generation, the lower (overwinter) and upper (summer) temperature optima and thermal limits of either species (e.g., for survival, flight, and reproduction), and phenological synchrony between the insects and their host plants (Mason et al. 2003, Williams 2010, Mathiasen et al. 2015a, b, Noronha and Mason 2017, Ortega-Ramos et al. 2022, Ewing et al. 2024). *P. chrysocephala* habitat suitability depended upon elevation above mean sea level, indicating that climatic factors and crop properties that are known to vary by elevation (Sundqvist et al. 2013, Rabia et al. 2022) are important in determining the potential range of *P. chrysocephala*. Habitat suitability for both *P. chrysocephala* and *B. viridescens* was also affected by precipitation metrics, *P. chrysocephala* by precipitation seasonality (i.e., the annual variation in monthly precipitation amounts, bio15) and *B. viridescens* by the mean monthly precipitation amount of the coldest quarter (bio19). Precipitation and its interactions with temperature influence insect survival, flight, reproduction, and development, as well as host plant fitness (Huberty and Denno 2004, Chown et al. 2011, Skendžić et al. 2021b), and there is evidence of such effects of precipitation on *P. chrysocephala* and *B. viridescens* [e.g., a positive effect of humidity on oviposition in *P. chrysocephala* and on dispersal and development in *B. viridescens*, and a negative effect of moisture on overwintering survival of *P. chrysocephala* larvae (reviewed in Mason et al. 2003, Williams 2010, Mathiasen et al. 2015a, Noronha and Mason 2017)]. In general, excessive precipitation/moisture can directly drown insects, impair flight, and physically interfere with overwintering processes, while too little moisture can reduce plant fitness and alter their susceptibility to impacts by herbivorous insects, and lead to decreased survival across life stages via water loss (Huberty and Denno 2004, Chown et al. 2011, Skendžić et al. 2021b).

Elevated CO_2_ levels driving climate change are implicated in both increasing global surface temperature and altered precipitation patterns (IPCC 2023), and thus our predictions for *P. chrysocephala* and *B. viridescens* under either SSP and time period reflect how temperature and precipitation are projected to influence the future potential ranges of these insects. It is important to consider, however, that insects may not exhibit predictable responses to shifting environmental conditions (Hortal et al. 2015, Lehmann et al. 2020, Skendžić et al. 2021b), and that projections of oilseed rape cultivation under climate change were not included in our models. Furthermore, climate change projections are themselves are subject to uncertainty (IPCC 2023), and it is unclear how climate change-induced environmental shifts will manifest at fine scales (e.g., at the crop field or plant level) (Lehmann et al. 2020, Skendžić et al. 2021b). Nevertheless, SDMs are some of the best tools available for forecasting how the overall species distributions of insects, such as *P. chrysocephala* and *B. viridescens*, may shift under climate change according to their environmental tolerances (Skendžić et al. 2021a, Gómez‑Undiano et al. 2022).

While much of North America was forecasted to become increasingly suitable for *P. chrysocephala* under climate change, westward spread of *B. viridescens* beyond the western Great Lakes region and across central North America will depend upon the degree of connectivity among patches of suitable habitat in the Canadian Prairies and Great Plains (USA) regions. These patches and the connecting landscape were projected to increase in suitability for *B. viridescens* by 2041 to 2070 under both climate change scenarios, indicating that risk to canola cultivation in central North America may increase over time if *B. viridescens* can disperse between suitable habitat patches. *Brassicogethes aeneus*, a close relative of *B. viridescens*, is thought to fly great distances (surpassing 13.5 km) from overwintering locations to oilseed rape crops (Taimr et al. 1967, Mauchline et al. 2017), suggesting that *B. viridescens* is likely also capable of long-distance dispersal across a landscape of fragmented habitat suitability.

While not included in our models, phenological synchrony between univoltine *P. chrysocephala* and *B. viridescens* populations and oilseed rape/canola crops in the current and projected ranges of either species will be crucial in the realization of their future distributions. In Europe, oilseed rape cultivation consists mainly of late summer/autumn-sown crops (WOSR) while in central North America, canola is primarily sown in spring (SOSR) (Kirkegaard et al. 2021, Canola Council of Canada 2024, U.S. Canola Association 2024). In Europe, *P. chrysocephala* recruit to feed and oviposit on emergent WOSR plants and overwinter as larvae inside their stems, only overwintering as adults when conditions are mild (Mathiasen et al. 2015a, Ortega-Ramos et al. 2022). *P. chrysocephala* is not reported to affect SOSR crops where they are grown in Europe (Williams 2010, Li et al. 2024), and thus it seems unlikely that *P. chrysocephala* would establish on SOSR crops in central North America. Additionally, the persistence of *P. chrysocephala* on SOSR in central North America would depend upon the availability of post-harvest stubble and non-crop Brassicaceae stems for overwintering. In eastern and southern North America and in the northwestern USA; however, where warmer winters allow for the cultivation of WOSR (Kirkegaard et al. 2021, Page et al. 2021, U.S. Canola Association 2024), the establishment potential of *P. chrysocephala* is probably greater than in central North America (i.e., due to theoretical synchrony between *P. chrysocephala* and WOSR). If *P. chrysocephala* were to become established in WOSR-growing regions of North America, where suitable habitat is broadly distributed and forecasted to increase under future climate change, impacts to crops could be severe. Our habitat suitability predictions for central North America more accurately represent risk from *B. viridescens*, as unlike *P. chrysocephala*, *B. viridescens* is adapted to SOSR phenology in Europe and eastern North America (Mason et al. 2003, Noronha and Mason 2017). Accordingly, *B. viridescens* poses less of a threat to WOSR where it is grown in North America. Although *B. viridescens* has the potential to cause major damage to SOSR crops in central North America, competition with existing pests such as *Lygus* bugs (Hemiptera: Miridae) and cabbage seedpod weevil, *Ceutorhynchus obstrictus* (Coleoptera: Curculionidae), that also feed upon SOSR reproductive tissues (Cárcamo et al. 2001, Otani and Cárcamo 2011, Cornelsen et al. 2023) could affect the ability of *B. viridescens* to establish in the region. Finally, because *B. viridescens* overwinters in the adult stage within leaf litter, practical management of *B. viridescens* should consider the benefits (e.g., conserving natural enemy communities) and potential costs (i.e., increased *B. viridescens* overwintering habitat) of maintaining border and hedgerow vegetation on affected fields (Williams 2010, Noronha and Mason 2017).

IPM of *P. chrysocephala* and *B. viridescens*, if either species becomes widespread in North American canola-growing regions, will almost certainly include neonicotinoid (for *P. chrysocephala*) and foliar (e.g., pyrethroid and carbamate) chemical insecticides (Noronha and Mason 2017, Ortega-Ramos et al. 2022, Cornelsen et al. 2023, Li et al. 2024). The most effective line of defense against flea beetles such as *P. chrysocephala* in Europe and *Phyllotreta* spp. in North America is sowing seed treated with systemic neonicotinoid insecticides (Ortega-Ramos et al. 2022, Cornelsen et al. 2023, Li et al. 2024). In Europe, a 2018 neonicotinoid ban released populations of *P. chrysocephala* from this previously effective control, and a compensatory increase in application of pyrethroid foliar insecticides led to the rapid development of pyrethroid resistance in this species (Ortega-Ramos et al. 2022, Li et al. 2024). Any future introductions of *P. chrysocephala* to North America are thus increasingly likely to include pyrethroid-resistant individuals, which would complicate the management of this species if it became widely established. While no insecticide resistance has been reported for *B. viridescens*, *B. aeneus* has developed resistance to pyrethroid foliar insecticides in Europe (Williams 2010, Noronha and Mason 2017), and thus future emergence of resistance in *B. viridescens* is not improbable. In addition to proactive risk assessment and monitoring, which our projections of habitat suitability for *P. chrysocephala* and *B. viridescens* will facilitate, we suggest that future work focuses on potential biopesticides that may be deployed against these pests (e.g., see Ortega-Ramos et al. 2022, Price et al. 2023) if they do evade detection and eradication and establish in previously unaffected canola-growing regions of North America.

## Supplementary Material

Supplementary material is available at *Journal of Economic Entomology* online.

toaf071_suppl_Supplementary_Material
