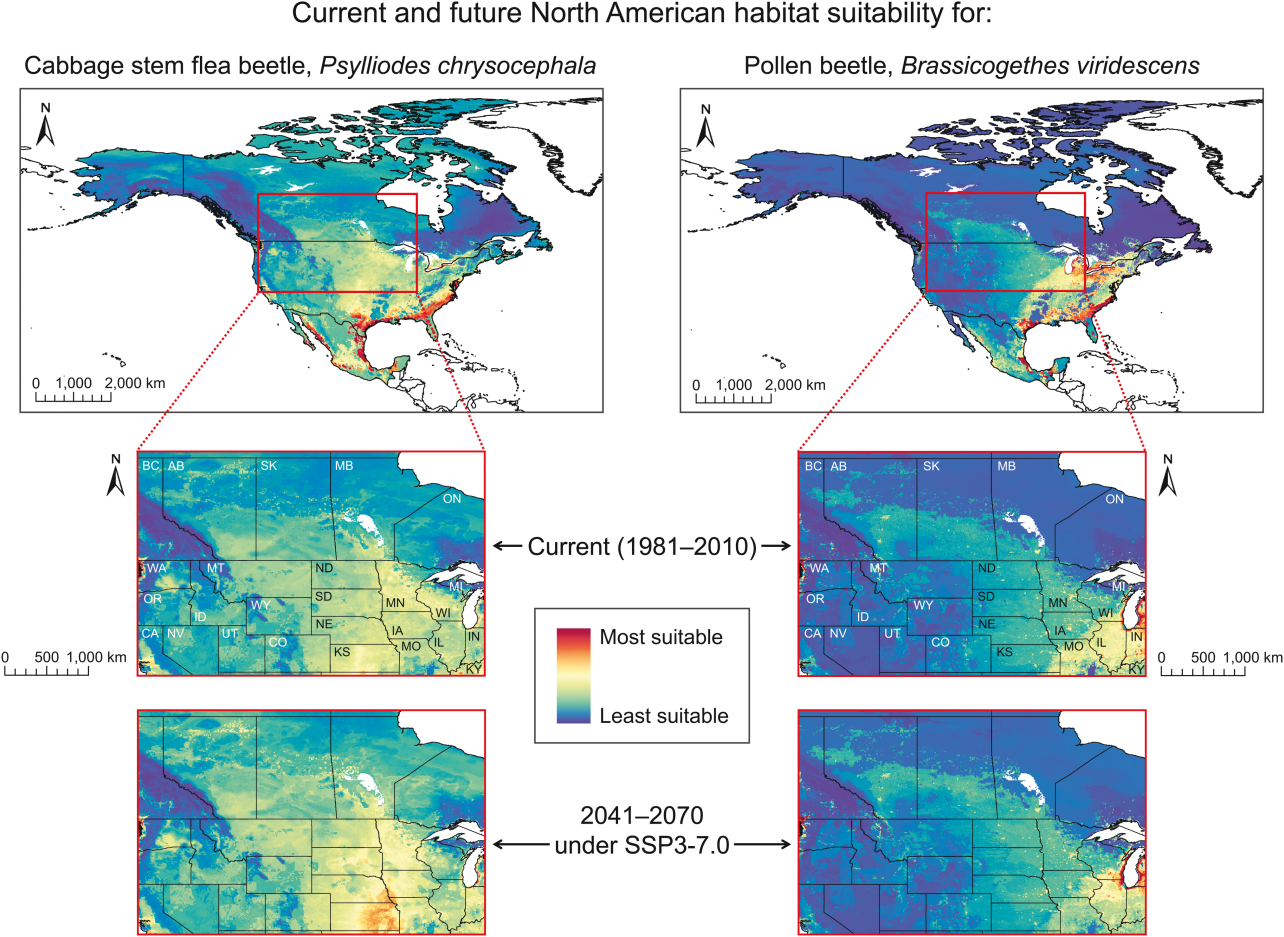# Correction to: Evaluating the establishment potential of cabbage stem flea beetle (Coleoptera: Chrysomelidae) and pollen beetle (Coleoptera: Nitidulidae) in canola-growing regions of North America using ensemble species distribution models

**DOI:** 10.1093/jee/toaf118

**Published:** 2025-05-14

This is a **correction** to:

Debra L Wertman, Vivek Srivastava, Tyler J Wist, Evaluating the establishment potential of cabbage stem flea beetle (Coleoptera: Chrysomelidae) and pollen beetle (Coleoptera: Nitidulidae) in canola-growing regions of North America using ensemble species distribution models, *Journal of Economic Entomology*, 2025;, toaf071, https://doi.org/10.1093/jee/toaf071

In the originally published version of this manuscript, the graphical abstract was erroneously duplicated as Figure 7. The paper has been updated with the correct Figure 7 and a higher resolution graphical abstract.

Figure 7:


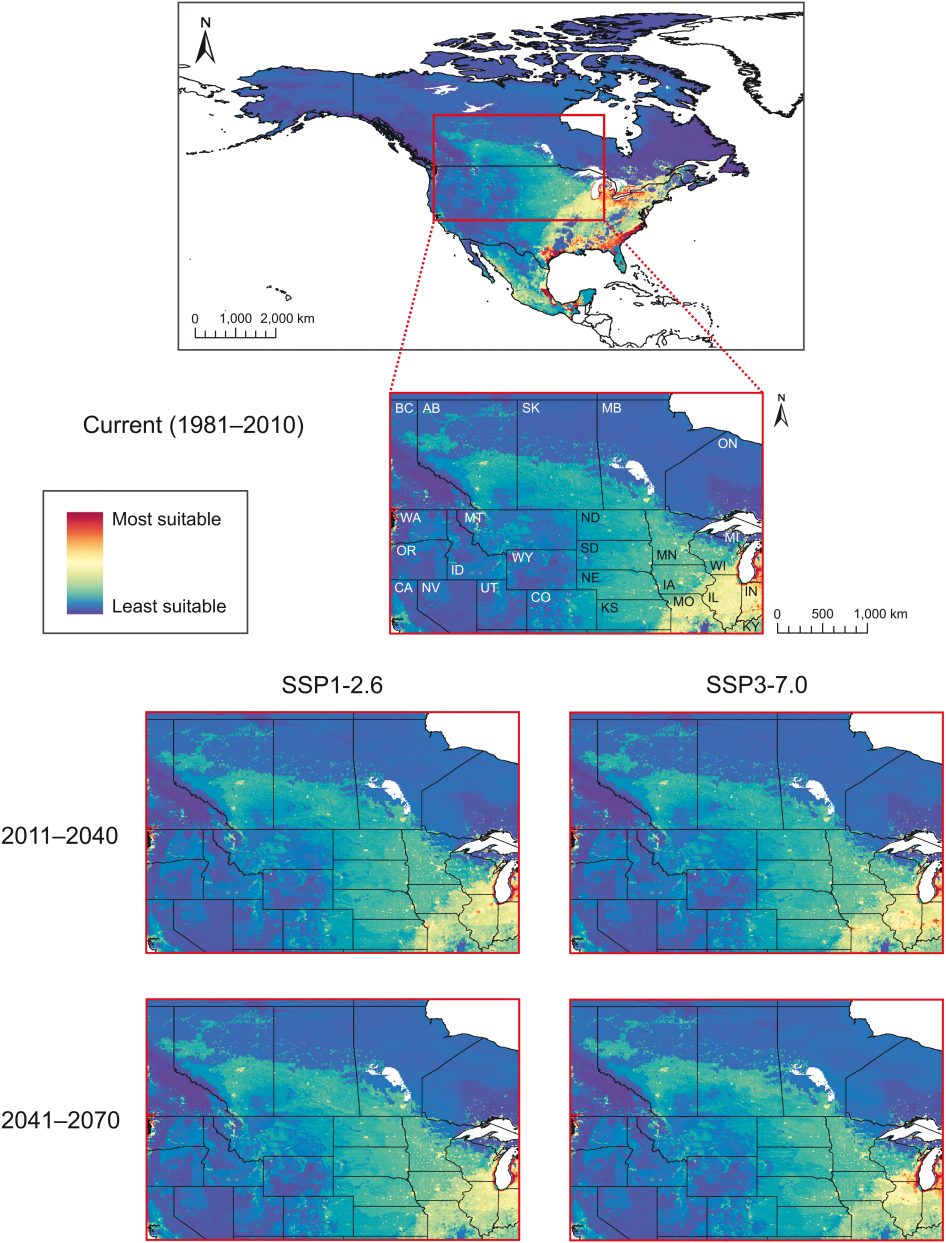


Graphical abstract