# Supplementary material for: Evaluating the establishment potential of cabbage stem flea beetle (Coleoptera: Chrysomelidae) and pollen beetle (Coleoptera: Nitidulidae) in canola-growing regions of North America using ensemble species distribution models
Source: J Econ Entomol. 2025 Apr 16;118(3):1281–96. doi: 10.1093/jee/toaf071 (PMC12167857; doi:10.1093/jee/toaf071)
Supplement: toaf071_suppl_Supplementary_Material [file toaf071_suppl_supplementary_material.pdf]

Evaluating the establishment potential of cabbage stem flea beetle (Coleoptera: Chrysomelidae) and pollen beetle (Coleoptera: Nitidulidae) in canola-growing regions of North America using ensemble species distribution models

Debra L. Wertman[1,2], Vivek Srivastava[1,2], and Tyler J. Wist[1]

[1] Saskatoon Research and Development Centre, Agriculture and Agri-Food Canada
[2] Department of Forest and Conservation Sciences, University of British Columbia

Supplemental Tables

Table S1. Environmental variables selected for modelling suitable habitat for cabbage stem flea beetle, *Psylliodes chrysocephala* (12 variables), and pollen beetle, *Brassicogethes viridescens* (11 variables), and biological justification for including each variable in the respective ensemble species distribution models (SDMs). The original resolution of each variable was 1 km.

Variable	Full name	Description	Unit	Temporal extent	Justification	Data source	Applicable species
bio2	Mean diurnal air temperature range	The mean diurnal range of temperatures averaged over 1 year	°C	1981-2010; 2011–2040; 2041–2070	Temperature affects overwintering and summer survival, flight and feeding activity, reproduction and development rates, and phenological synchrony with host plants in herbivorous insects (Buckley et al. 2017, Lehmann et al. 2020, Skendžić et al. 2021b)	Chelsa database version 2.1 (Karger et al. 2017, 2021)	*Psylliodes chrysocephala*; *Brassicogethes viridescens*
bio7	Annual range of air temperature	The difference between the maximum temperature of warmest month and the minimum temperature of coldest month	°C				*Psylliodes chrysocephala*; *Brassicogethes viridescens*
bio8	Mean daily mean air temperatures of the wettest quarter	The mean daily air temperatures of the wettest quarter of the year (to the nearest month)	°C		Known interaction effects of temperature and precipitation on survival, flight, reproduction, and development in herbivorous insects, and on host plant fitness (Chown et al. 2011, Skendžić et al. 2021b)		*Psylliodes chrysocephala*; *Brassicogethes viridescens*

Variable	Full name	Description	Unit	Temporal extent	Justification	Data source	Applicable species
bio9	Mean daily mean air temperatures of the driest quarter	The mean daily air temperatures of the driest quarter of the year (to the nearest month)	°C	(Continued)	(Continued)	(Continued)	*Psylliodes chrysocephala*
bio14	Precipitation of the driest month	The precipitation of the driest month	kg/m^2 per month		Precipitation and water availability affect survival, flight, reproduction, and development in herbivorous insects, and host plant fitness (Huberty and Denno 2004, Chown et al. 2011, Skendžić et al. 2021b)		*Brassicogethes viridescens*
bio15	Precipitation seasonality	The annual variation in monthly precipitation totals (coefficient of variation, i.e., the standard deviation of the monthly precipitation estimates expressed as a percentage of the annual mean of those estimates)	kg/m^2				*Psylliodes chrysocephala*
bio18	Mean monthly precipitation amount of the warmest quarter	The mean monthly precipitation amount of the warmest quarter of the year (to the nearest month)	kg/m^2 per month		Known interaction effects of temperature and precipitation on survival, flight, reproduction, and development in herbivorous insects, and on host plant fitness (Chown et al. 2011, Skendžić et al. 2021b)		*Psylliodes chrysocephala*; *Brassicogethes viridescens*
bio19	Mean monthly precipitation amount of the coldest quarter	The mean monthly precipitation amount of the coldest quarter of the year (to the nearest month)	kg/m^2 per month				*Psylliodes chrysocephala*; *Brassicogethes viridescens*

Variable	Full name	Description	Unit	Temporal extent	Justification	Data source	Applicable species
CD	Crop dominance	Global cropland cover by class	N/A (categorical)	2010	Feeding and reproduction of both target species are specialized on Brassicaceae, including *Brassica* spp. such as oilseed rape (Mason et al. 2003, Li et al. 2024), which is captured by this global cropland variable	NASA MEaSUREs Global Food Security Support Analysis Data (GFSAD; Thenkabail et al. 2016)	*Psylliodes chrysocephala*; *Brassicogethes viridescens*
elev	Elevation	Elevation above mean sea level (derived from the GTOPO30 DEM)	m	NA	Elevation is linked to local climate patterns and crop properties, which affect herbivorous insect distribution (Sundqvist et al. 2013, Skendžić et al. 2021b, Rabia et al. 2022)	United States Geological Survey (USGS 2018)	*Psylliodes chrysocephala*; *Brassicogethes viridescens*
HII	Human Influence Index	Composite of nine global human impact layers including population density, land use, infrastructure, and land access	N/A	1995–2004	Known relationships between human activity and invasion pathways, and likelihood of insect species introduction and establishment (Gippet et al. 2019, Skendžić et al. 2021a, Fenn-Moltu et al. 2023)	NASA Socioeconomic Data and Applications Center (SEDAC) [WCS (Wildlife Conservation Society)/CIESIN (Center for International Earth Science Information Network, Columbia University) 2005]	*Psylliodes chrysocephala*; *Brassicogethes viridescens*
kg2	Köppen-Geiger climate classification	Köppen Geiger climate classification (Peel et al. 2007)	N/A (categorical)	1981-2010; 2011–2040; 2041–2070	Regional climate affects insect distribution and fitness (Skendžić et al. 2021b)	Chelsa database version 2.1 (Karger et al. 2017, 2021)	*Psylliodes chrysocephala*; *Brassicogethes viridescens*

Variable	Full name	Description	Unit	Temporal extent	Justification	Data source	Applicable species
NA	Nutrient availability	Soil characteristics including texture, organic carbon, pH, and total exchangeable bases	N/A	2008	Nutrient availability affects host plant growth and vigour, and influences herbivorous insect feeding activity (Sundqvist et al. 2013, Kirkegaard et al. 2021, Skendžić et al. 2021b, Rabia et al. 2022)	Harmonized World Soil Database v 1.2 (Fisher et al. 2008)	*Psylliodes chrysocephala*; *Brassicogethes viridescens*

Table S2. Variance inflation factor (VIF) scores for 36 linear predictor environmental variables that were considered for modelling suitable habitat for cabbage stem flea beetle, *Psylliodes chrysocephala*, and pollen beetle, *Brassicogethes viridescens*. Only variables with global coverage, VIF scores of < 10 (after exclusion of collinear variables), and Pearson correlation coefficients (*r*) of |< 0.70| (see Figure S1) were selected for inclusion in either ensemble species distribution model (SDM). See Table S1 and **Materials and Methods: *Environmental variables for model development*** in the main text for variable source and selection details.

Species	Variable	VIF before exclusion of collinear variables	VIF after exclusion of collinear variables
Psylliodes chrysocephala	bio1	597.08	-
	bio2	52.56	6.92
	bio3	80.54	-
	bio4	1417.84	-
	bio5	Inf	-
	bio6	Inf	-
	bio7	Inf	6.15
	bio8	4.06	3.44
	bio9	10.79	4.20
	bio10	753.45	-
	bio11	2742.67	-
	bio12	150.34	-
	bio13	117.78	-
	bio14	119.39	-
	bio15	6.57	2.29
	bio16	268.78	-
	bio17	166.38	-
	bio18	42.80	2.24
	bio19	35.19	2.13
	CD	1.83	1.65
	elev	3.95	3.11
	fcf	6.45	3.15
	fgd	5.32	3.07
	gdd0	9898.90	-
	gdd5	37504.24	-
	gdd10	19549.70	-
	gsp	20.38	-
	gst	4.76	2.54
	HII	2.69	2.17
	kg2	3.71	2.23
	NA	3.48	3.09
	ngd0	380.51	-
	ngd5	1317.50	-
	ngd10	614.00	-
	npp	11.04	-
	scd	183.91	-

Species	Variable	VIF before exclusion of collinear variables	VIF after exclusion of collinear variables
Brassicogethes viridescens	bio1	556.62	-
	bio2	53.98	6.29
	bio3	81.87	-
	bio4	1312.38	-
	bio5	Inf	-
	bio6	Inf	-
	bio7	Inf	4.46
	bio8	3.91	3.59
	bio9	10.76	-
	bio10	671.07	-
	bio11	2687.80	-
	bio12	130.77	-
	bio13	88.19	-
	bio14	115.83	5.29
	bio15	7.42	3.75
	bio16	199.28	-
	bio17	150.41	-
	bio18	32.17	5.91
	bio19	27.58	4.43
	CD	1.82	1.92
	elev	4.15	3.32
	fcf	6.21	2.83
	fgd	5.41	3.53
	gdd0	10428.99	-
	gdd5	23708.25	-
	gdd10	12011.65	4.78
	gsp	18.86	4.40
	gst	4.89	2.53
	HII	2.68	2.47
	kg2	3.66	3.30
	NA	3.32	3.03
	ngd0	353.19	-
	ngd5	1160.71	-
	ngd10	318.22	-
	npp	12.12	-
	scd	174.40	-

Supplemental Figures

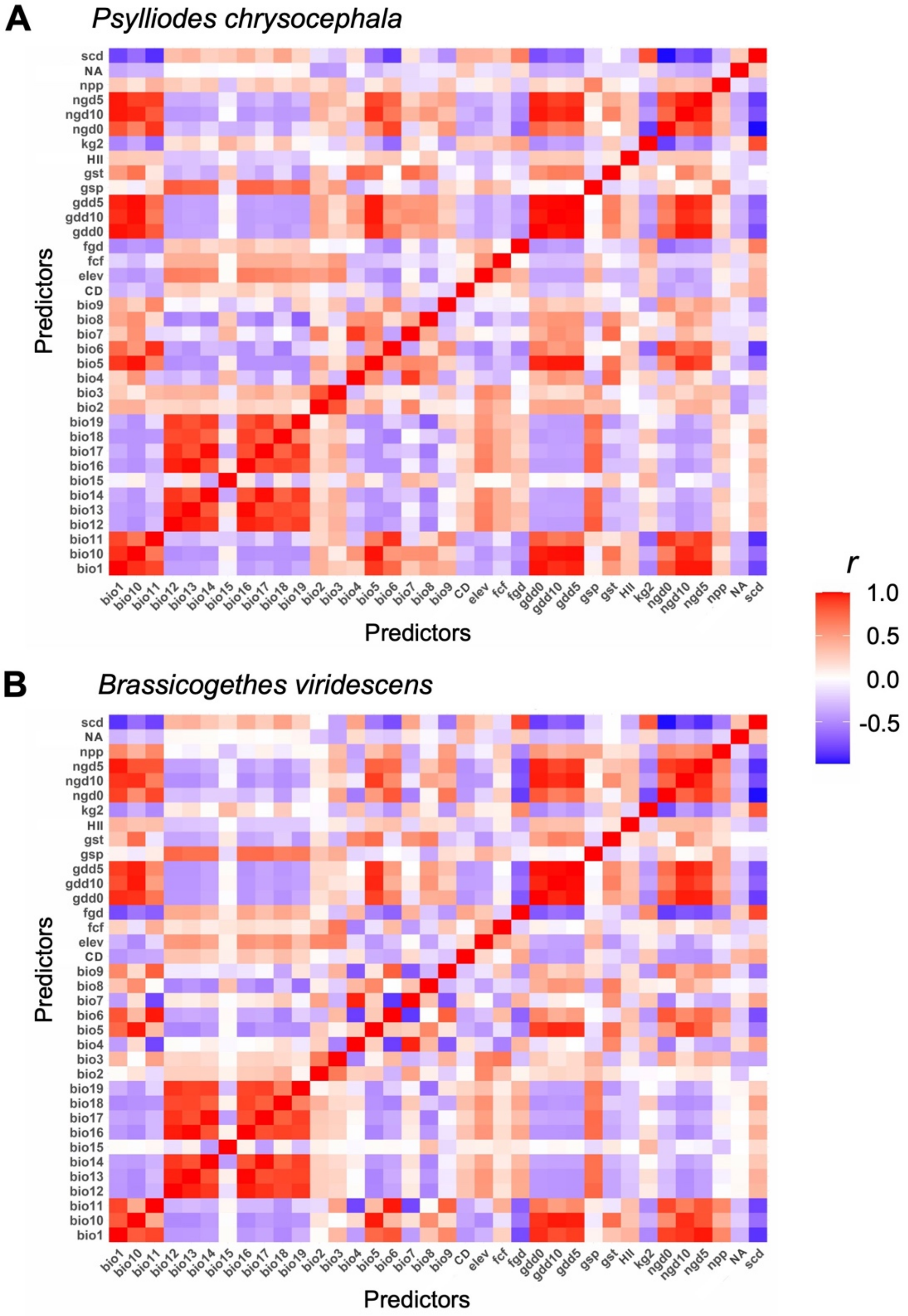

Figure S1. Figures showing pairwise correlations among 36 linear predictor environmental variables that were considered for modelling suitable habitat for A: cabbage stem flea beetle, *Psylliodes chrysocephala*, and B: pollen beetle, *Brassicogethes viridescens*. Only variables with global coverage, Pearson correlation coefficients (r) of |< 0.70|, and variance inflation factor (VIF) scores of < 10 (see Table S2) were selected for inclusion in either ensemble species distribution model (SDM). See Table S1 and **Materials and Methods: *Environmental variables for model development*** in the main text for variable source and selection details.

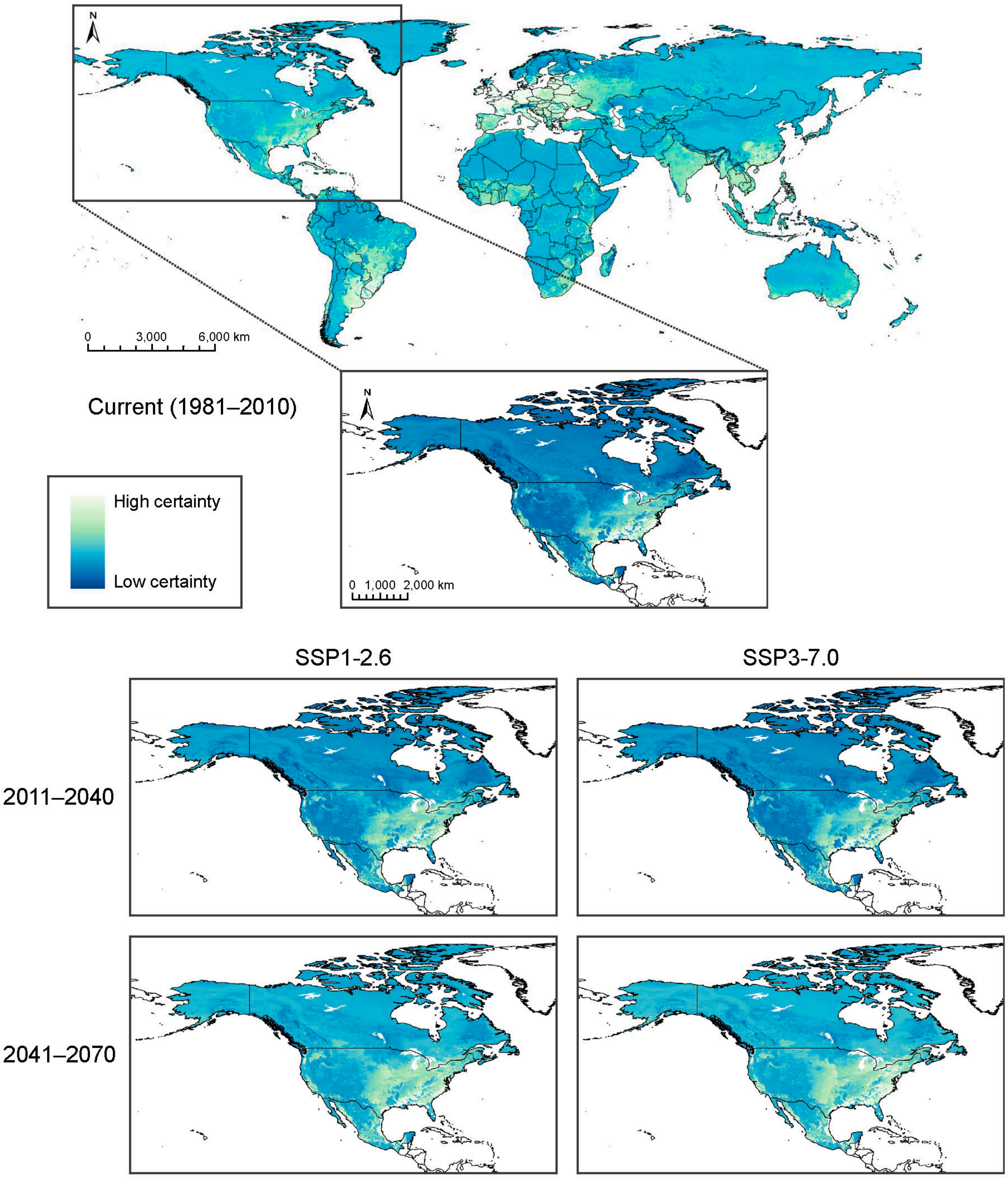

Figure S2. Sensitivity maps showing range of prediction certainty [i.e., coefficient of variation (SD/mean) of probabilities] for a cabbage stem flea beetle, *Psylliodes chrysocephala*, weighted-means ensemble species distribution model (SDM) projected across current conditions (1981–2010; global at 10 km resolution, North America at 5 km resolution) and, for North America (at 5 km resolution), future climatic scenarios [SSP1-2.6 and SSP3-7.0, Shared Socio-economic Pathways (SSPs) from the Intergovernmental Panel on Climate Change (IPCC)] at two time periods (2011–2040 and 2041–2070).

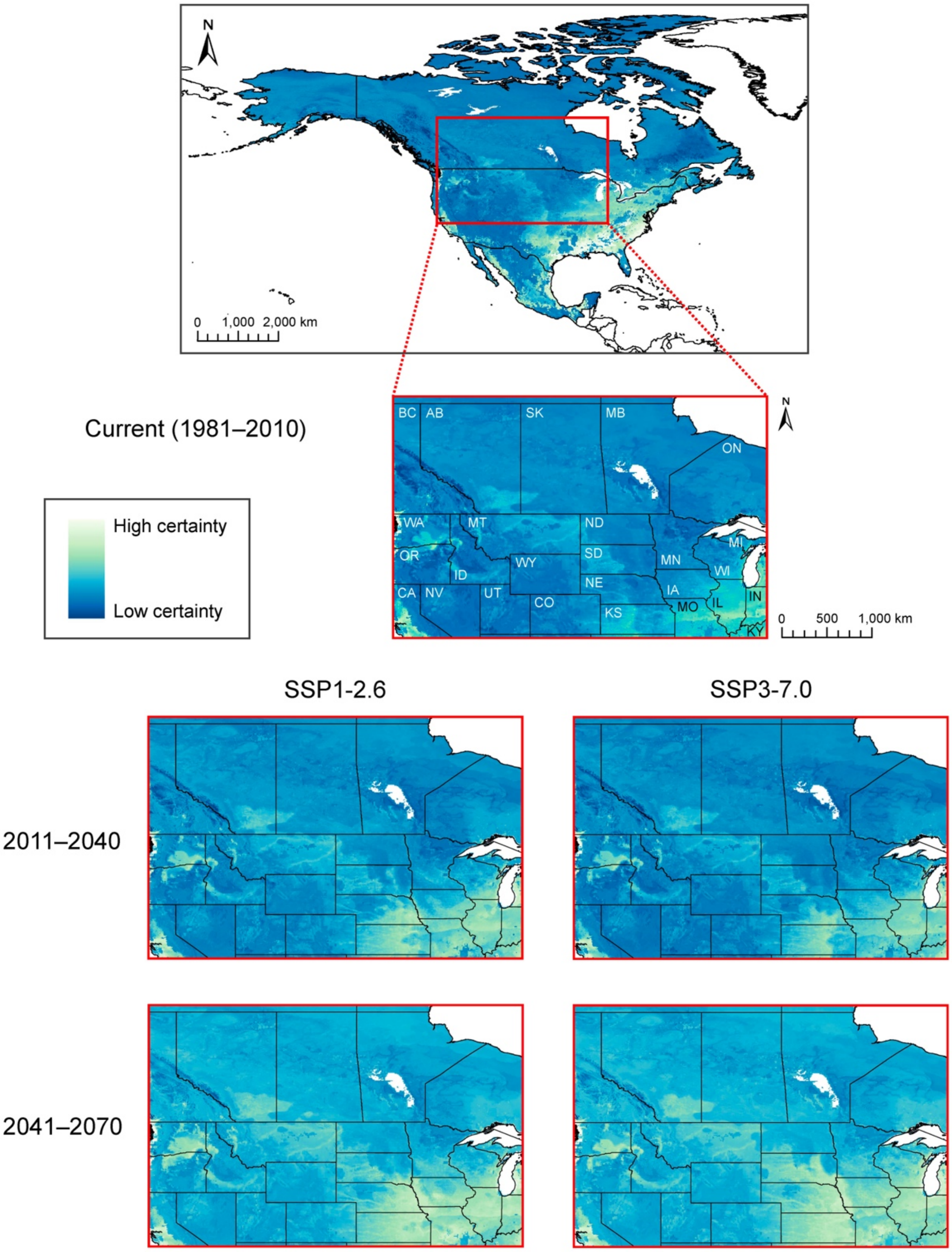

Figure S3. Sensitivity maps (at 5 km resolution) showing range of estimate certainty [i.e., coefficient of variation (SD/mean) of probabilities] for the cabbage stem flea beetle, *Psylliodes chrysocephala*, weighted-means ensemble species distribution model (SDM) projected across North America [current condition map of North America from Fig. S2; insets show central North America, including the Canadian Prairies and Great Plains (USA)] under current conditions (1981–2010) and future climatic scenarios [SSP1-2.6 and SSP3-7.0, Shared Socio-economic Pathways (SSPs) from the Intergovernmental Panel on Climate Change (IPCC)] at two time periods (2011–2040 and 2041–2070).

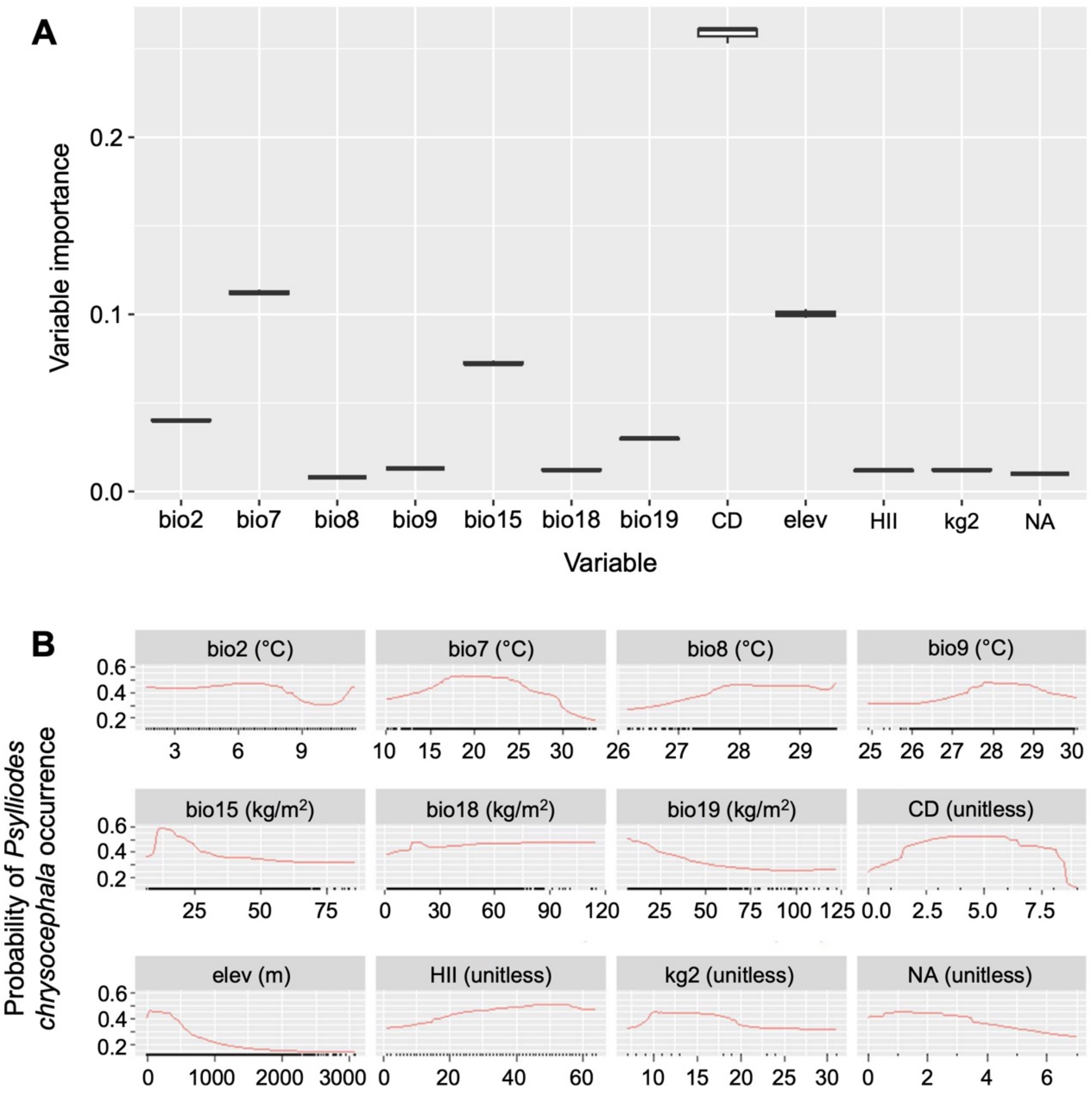

Figure S4. A: Variable importance for the weighted-means ensemble model describing habitat suitability for cabbage stem flea beetle, *Psylliodes chrysocephala*. Horizontal lines show median variable importance, upper and lower boxes indicate the 75th and 25th quartiles, respectively, and vertical lines represent maximum and minimum values. B: Response curves (red lines) showing the relationship between each environmental variable and ensemble model predictions for *P. chrysocephala* habitat suitability. See Table 1 (**Methods**: ***Environmental variables for model development***) for variable definitions, units, and data sources.

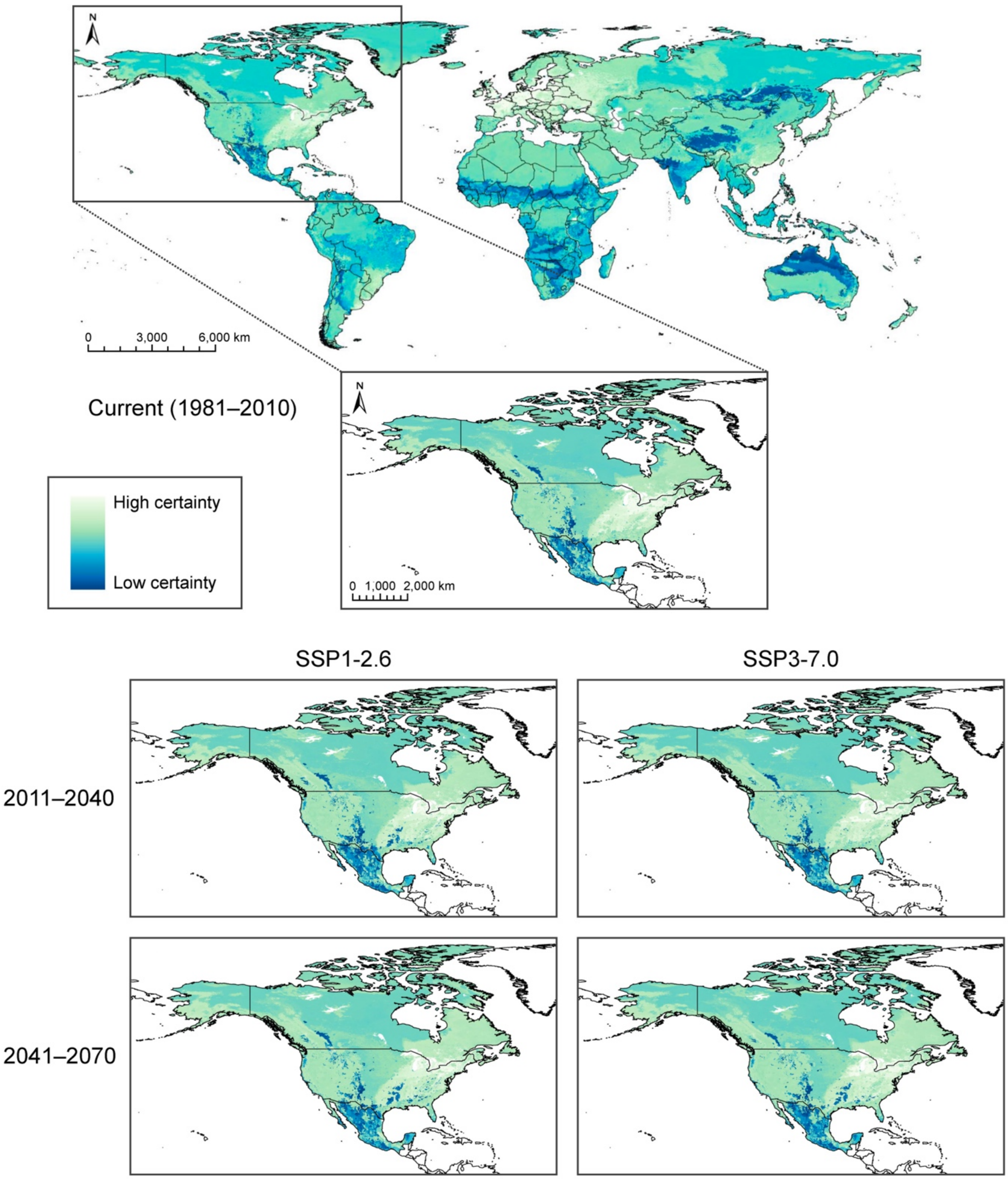

Figure S5. Sensitivity maps showing range of estimate certainty [i.e., coefficient of variation (SD/mean) of probabilities] for a pollen beetle, *Brassicogethes viridescens*, weighted-means ensemble species distribution model (SDM) projected across current conditions (1981–2010; global at 10 km resolution, North America at 5 km resolution) and, for North America (at 5 km resolution), future climatic scenarios [SSP1-2.6 and SSP3-7.0, Shared Socio-economic Pathways (SSPs) from the Intergovernmental Panel on Climate Change (IPCC)] at two time periods (2011–2040 and 2041–2070).

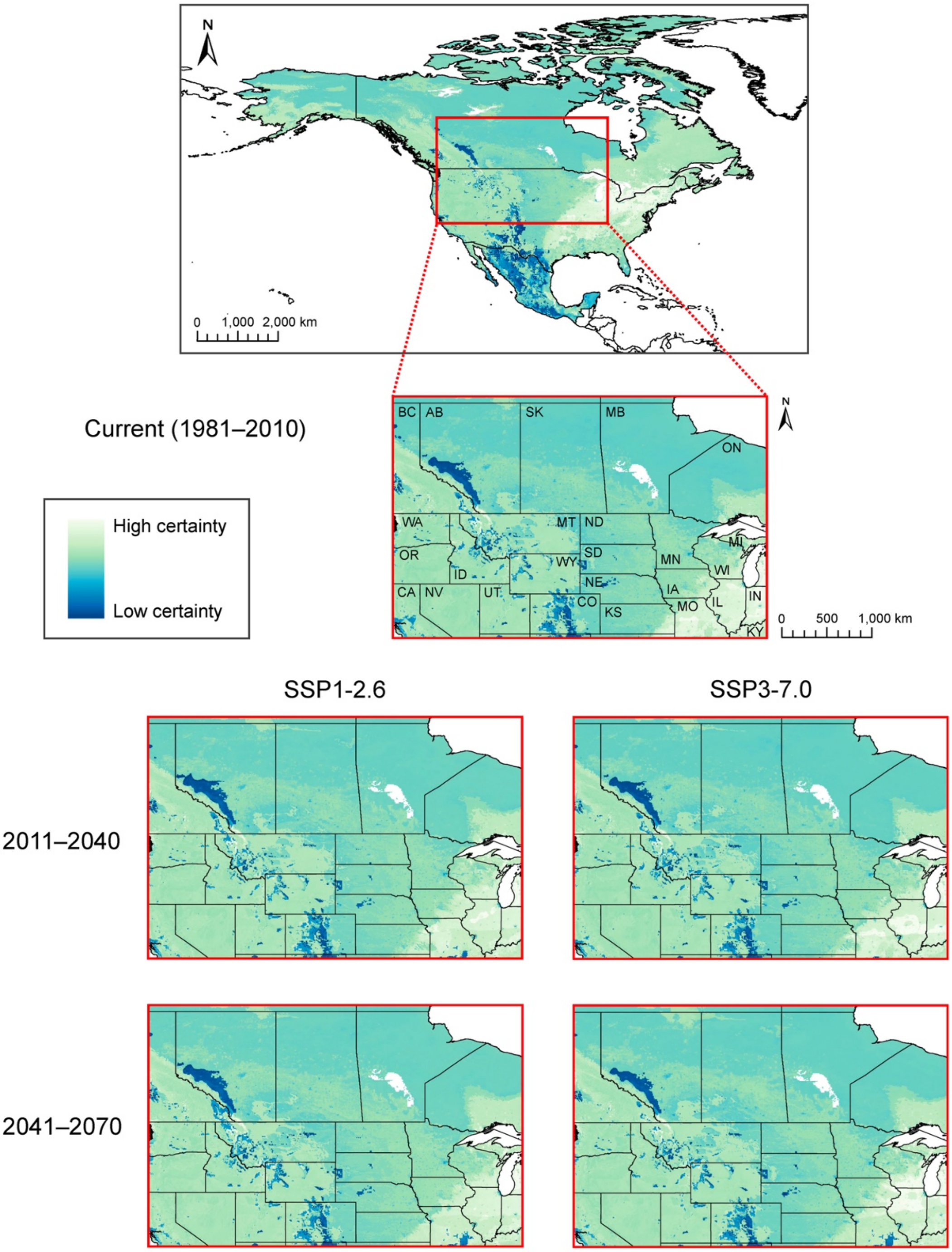

Figure S6. Sensitivity maps (at 5 km resolution) showing range of prediction certainty [i.e., coefficient of variation (SD/mean) of probabilities] for the pollen beetle, *Brassicogethes viridescens*, weighted-means ensemble species distribution model (SDM) projected across North America [current condition map of North America from Fig. S5; insets show central North America, including the Canadian Prairies and Great Plains (USA)] under current conditions (1981–2010) and future climatic scenarios [SSP1-2.6 and SSP3-7.0, Shared Socio-economic Pathways (SSPs) from the Intergovernmental Panel on Climate Change (IPCC)] at two time periods (2011–2040 and 2041–2070).

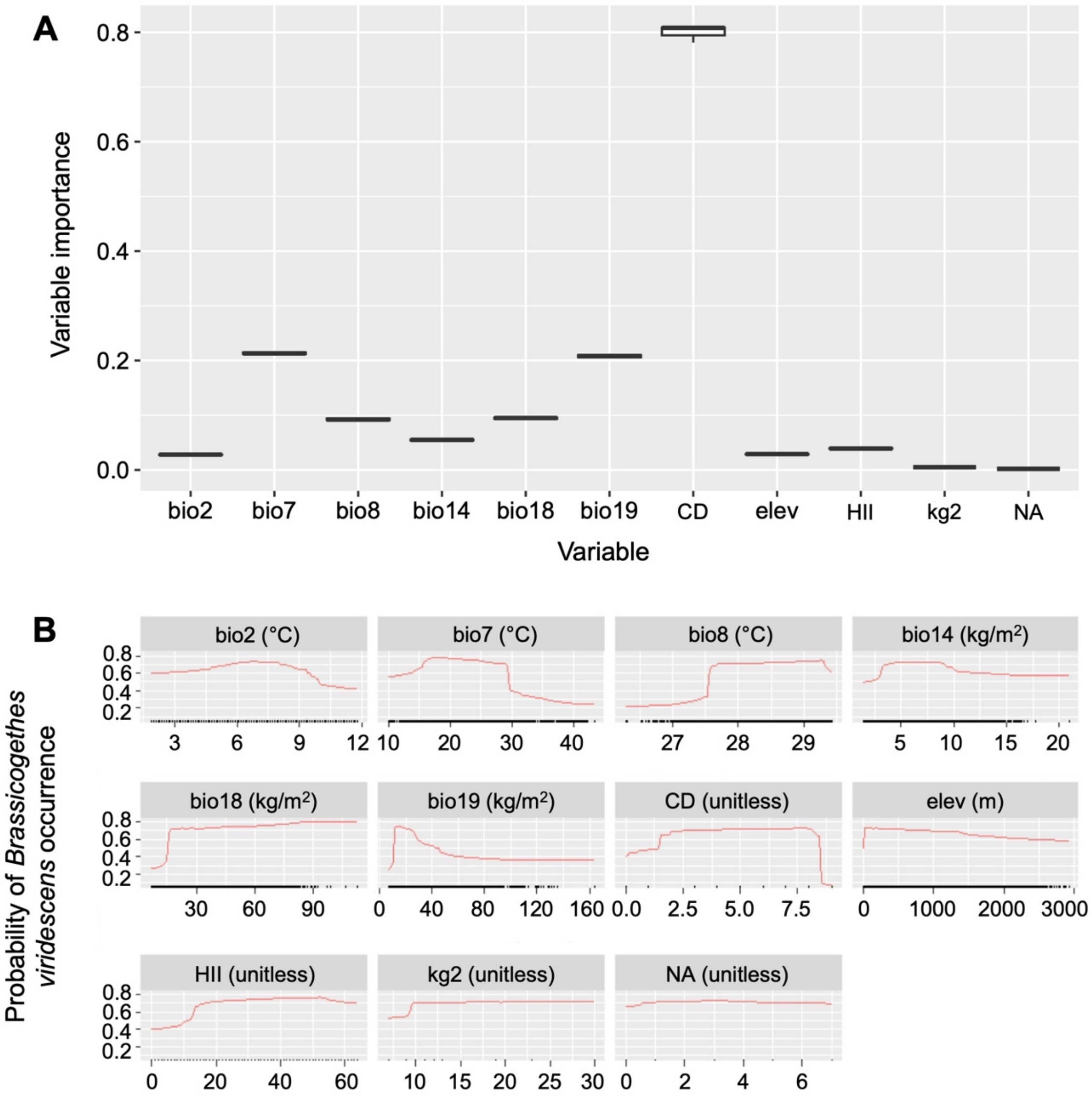

Figure S7. A: Variable importance for the weighted-means ensemble model describing pollen beetle, *Brassicogethes viridescens*, habitat suitability. Horizontal lines indicate median variable importance, upper and lower boxes represent the 75th and 25th quartiles, respectively, and vertical lines show maximum and minimum values. B: Response curves (red lines) illustrating the relationship between each environmental variable and ensemble model predictions for *B. viridescens* habitat suitability. See Table 1 (**Methods**: ***Environmental variables for model development***) for variable definitions, units, and data sources.

Supplemental References

Buckley LB, Arakaki AJ, Cannistra AF, et al. 2017. Insect development, thermal plasticity and fitness implications in changing, seasonal environments. Integr. Comp. Biol. 57(5):988–998. https://doi.org/10.1093/icb/icx032.

Chown SL, Sørensen JG, Terblanche JS. 2011. Water loss in insects: An environmental change perspective. J. Insect Physiol. 57(8):1070–1084. https://doi.org/10.1016/j.jinsphys.2011.05.004.

Fenn-Moltu G, Ollier S, Caton B, et al. 2023. Alien insect dispersal mediated by the global movement of commodities. Ecol. Appl. 33(1):e2721. https://doi.org/10.1002/eap.2721.

Fischer G, Nachtergaele F, Prieler S, et al. 2008. Global agro-ecological zones assessment for agriculture (GAEZ 2008). IIASA, Laxenburg, Austria and FAO, Rome, Italy. Available from: https://www.fao.org/soils-portal/data-hub/soil-maps-and-databases/harmonized-world-soil-database-v12/en/. Accessed 22 February 2024.

Gippet JM, Liebhold AM, Fenn-Moltu G, et al. 2019. Human-mediated dispersal in insects. Curr. Opin. Insect Sci. 35:96–102. https://doi.org/10.1016/j.cois.2019.07.005.

Huberty AF, Denno RF. 2004. Plant water stress and its consequences for herbivorous insects: A new synthesis. Ecology. 85(5):1383–1398. https://doi.org/10.1890/03-0352.

Karger DN, Conrad O, Böhner J, et al. 2017. Climatologies at high resolution for the earth’s land surface areas. Sci. Data. 4(1):170122. https://doi.org/10.1038/sdata.2017.122.

Karger DN, Conrad O, Böhner J, et al. 2021. Climatologies at high resolution for the earth’s land surface areas. EnviDat. https://doi.org/10.16904/envidat.228.v2.1. Accessed 22 February 2024.

Kirkegaard JA, Lilley JM, Berry PM, et al. 2021. Canola. In: Sadras VO, Calderini DF, editors. Crop physiology case histories for major crops. Academic Press/Elsevier Inc. p. 518–549. https://doi.org/10.1016/b978-0-12-819194-1.00017-7.

Lehmann P, Ammunét T, Barton M, et al. 2020. Complex responses of global insect pests to climate warming. Front. Ecol. Environ. 18(3):141–150. https://doi.org/10.1002/fee.2160.

Li Z, Costamagna AC, Beran F, et al. 2024. Biology, ecology, and management of flea beetles in *Brassica* crops. Annu. Rev. Entomol. 69(1):199–217. https://doi.org/10.1146/annurev-ento-033023-015753.

Mason PG, Olfert O, Sluchinski L, et al. 2003. Actual and potential distribution of an invasive canola pest, Meligethes viridescens (Coleoptera: Nitidulidae), in Canada. Can. Entomol. 135(3):405–413. https://doi.org/10.4039/n02-046.

Rabia AH, Neupane J, Lin Z, et al. 2022. Principles and applications of topography in precision agriculture. In: Sparks DL, editor. Advances in agronomy. Vol. 171. Academic Press/Elsevier Inc. p. 143–189. https://doi.org/10.1016/bs.agron.2021.08.005.

Skendžić S, Zovko M, Živković IP, et al. 2021a. Effect of climate change on introduced and native agricultural invasive insect pests in Europe. Insects. 12(11):985. https://doi.org/10.3390/insects12110985.

Skendžić S, Zovko M, Živković IP, et al. 2021b. The impact of climate change on agricultural insect pests. Insects. 12(5):440. https://doi.org/10.3390/insects12050440.

Sundqvist MK, Sanders NJ, Wardle DA. 2013. Community and ecosystem responses to elevational gradients: Processes, mechanisms, and insights for global change. Annu. Rev. Ecol., Evol. Syst. 44(1):261–280. https://doi.org/10.1146/annurev-ecolsys-110512-135750.

Thenkabail P, Teluguntla P, Xiong J, et al. 2016. NASA MEaSUREs Global Food Security Support Analysis Data (GFSAD) Crop Dominance 2010 Global 1 km V001. NASA EOSDIS Land Processes DAAC. https://doi.org/10.5067/MEaSUREs/GFSAD/GFSAD1KCD.001. Accessed 22 February 2024.

USGS (United States Geological Survey). 2018. Global 30 arc-second elevation (GTOPO30). USGS EROS Archive. https://doi.org/10.5066/F7DF6PQS. Accessed 22 February 2024.

WCS (Wildlife Conservation Society)/CIESIN (Center for International Earth Science Information Network, Columbia University). 2005. Last of the Wild Project, Version 2, 2005 (LWP-2): Global Human Influence Index (HII) Dataset (Geographic). NASA Socioeconomic Data and Applications Center (SEDAC). https://doi.org/10.7927/H4BP00QC. Accessed 22 February 2024.
